# Designing Cyclic-Constrained Peptides to Inhibit Human Phosphoglycerate Dehydrogenase

**DOI:** 10.3390/molecules28176430

**Published:** 2023-09-04

**Authors:** Xiaoyu Qing, Qian Wang, Hanyu Xu, Pei Liu, Luhua Lai

**Affiliations:** 1 BNLMS, Peking-Tsinghua Center for Life Sciences, College of Chemistry and Molecular Engineering, Peking University, Beijing 100871, China; xiaoyuqing6601@gmail.com (X.Q.); hyxu_natalia@126.com (H.X.); peiliu9052@gmail.com (P.L.); 2 State Key Laboratory of Natural and Biomimetic Drugs, School of Pharmaceutical Sciences, Peking University, Beijing 100191, China; qian.wang@bjmu.edu.cn; 3 Center for Quantitative Biology, Academy for Advanced Interdisciplinary Studies, Peking University, Beijing 100871, China

**Keywords:** loop epitopes, cyclic-constrained peptides, obligate homo-oligomers, protein-protein modulators, structure-based peptide design and optimization

## Abstract

Although loop epitopes at protein-protein binding interfaces often play key roles in mediating oligomer formation and interaction specificity, their binding sites are underexplored as drug targets owing to their high flexibility, relatively few hot spots, and solvent accessibility. Prior attempts to develop molecules that mimic loop epitopes to disrupt protein oligomers have had limited success. In this study, we used structure-based approaches to design and optimize cyclic-constrained peptides based on loop epitopes at the human phosphoglycerate dehydrogenase (PHGDH) dimer interface, which is an obligate homo-dimer with activity strongly dependent on the oligomeric state. The experimental validations showed that these cyclic peptides inhibit PHGDH activity by directly binding to the dimer interface and disrupting the obligate homo-oligomer formation. Our results demonstrate that loop epitope derived cyclic peptides with rationally designed affinity-enhancing substitutions can modulate obligate protein homo-oligomers, which can be used to design peptide inhibitors for other seemingly intractable oligomeric proteins.

## 1. Introduction

Protein-protein interactions (PPIs) occurring between identical or non-identical chains to form homo- or hetero-oligomers are common and often crucial for protein activity [1,2]. Modulation of the oligomerization equilibrium has emerged as a potential therapeutic strategy that can be applied to many biological systems, such as cancer and viral infections [3,4,5,6,7]. Developing oligomerization-modulating compounds based on hot spots, which are the main contributors to binding affinity and PPI stability, has proven a valuable strategy [8,9,10]. However, hot spots tend to be highly conserved, with significant sequence and structural similarities among different proteins, both within and across protein families [11,12,13,14]. Given the high degree of similarity present in these hot spots, it remains challenging to design high affinity PPI modulators targeting these hot spots with sufficient specificity. In explorations of alternative approaches, loop epitopes have emerged as particularly interesting sites, since many protein complexes have loops present at the binding interfaces. These loop regions may play key roles in mediating oligomer formation and determining interaction specificity through mechanisms such as interface insertion/deletion or interface add-ons [15,16]. Prior studies have used various computational and experimental strategies to design chemical modulators mimicking loop epitopes or directly converting native loop structures into macrocycles or cyclic peptides to disrupt transitional, non-obligate hetero-oligomeric proteins [17,18,19]. However, the efficacy of these approaches for disrupting loop epitope-mediated homo-oligomers remains largely unexplored [20,21,22].

In this study, we designed cyclic-constrained peptides based on loop epitopes of the homo-dimer interface to inhibit its enzyme activity by disrupting PHGDH homo-dimerization. PHGDH is a key enzyme in the serine biosynthesis pathway in human cells, which supports several metabolic processes that are essential for the normal growth and survival of proliferating cells [23,24,25]. Because the abnormal growth and proliferation characteristic of various cancer cells is accompanied by increased PHGDH activity and expression, PHGDH has been considered a promising anti-cancer drug target. However, all previously reported PHGDH inhibitors suffered low in vitro or in vivo activity, and developing highly active and specific inhibitors remains challenging [26,27]. PHGDH belongs to the D-isomer specific 2-hydroxyacid dehydrogenase (2HADH) family, which contains a wide range of oxidoreductases that catalyze reductions of 2-keto acids to corresponding 2-hydroxy acids [28]. Though the sequence identities of these enzymes are only around 35%, their structures are highly conserved, and almost all 2HADH enzymes function as homo-dimers [28]. PHGDH is a type I phosphoglycerate dehydrogenase containing two additional regulatory domains that form a dimer and tetramer mixture in solution, although its catalytic unit (sPHGDH: 1-307) was solved as a homo-dimer [29,30]. The dimer interface of PHGDH is predominantly composed of a continuous epitope that includes a core helix region buried in the binding interfaces (N_102_SLSAAELTCGMIMCLAR_119_), followed by an interfacial loop referred to as the dimerization loop (Q_120_IPQATASMKDGKWERKKFMG_140_). Compared to the relatively druggable portions of the interface, like the core helix region that are more hydrophobic and lack apparent conformational changes upon binding, the dimerization loop is highly flexible, solvent accessible, and contains a larger number of polar and charged amino acids (11 out of 20 amino acids). These properties suggest that the dimerization loop may play an important role in mediating interaction specificity and, therefore, may be a good candidate for testing the feasibility of designing PPI modulators based on loop epitopes at the interfaces of obligate homo-oligomers. Here, we employed structure-based approaches to generate cyclic peptides based on the dimerization loop of PHGDH and optimized their activity and binding affinity through rationally designed substitutions that enhance side-chain interactions and hydrogen bond networks. The experimental validations showed that these cyclic peptides were capable of binding to the interface and disrupting the oligomerization equilibrium of PHGDH, thereby inhibiting its activity. Although the identified peptides are capable of disrupting oligomer formation, further optimization is necessary to improve their binding affinity and PHGDH inhibition potency. Our results demonstrate that, in addition to hot spot regions, loop epitopes at the interfaces of homo-oligomers can be used to guide the design of oligomerization modulating compounds.

## 2. Results

### 2.1. Cyclic-Constrained Peptides Design and Optimization

**Figure 1:**
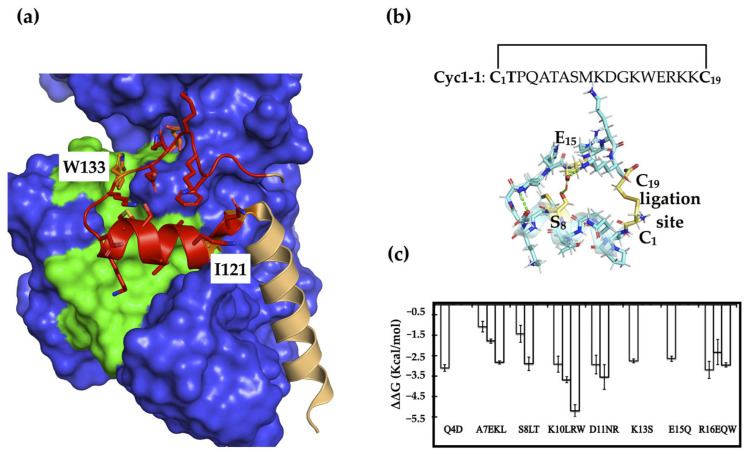
The dimer interface and constrained peptide design/optimization based on the dimerization loop. (**a**) The dimer interface of PHGDH is mainly composed of a core helix (light orange) and the dimerization loop (red) (PDB code: 2G76), the two hot spots including I121 and W133 are shown in orange. The binding surface of the loop is colored green, and the rest of the binding protein is colored blue. (**b**) A disulfide-cyclized peptide was constructed based on the dimerization loop through positions Q120 and F137 (corresponding to C1 and C19 in the cyclic peptide, colored as yellow). The other possible pair (S8-E15) for inserting a disulfide bridge is also colored in yellow, and the rest of the cyclic peptide is shown in light blue. (**c**) The most beneficial substitutions for enhancing the affinity of this cyclic peptide were predicted using the flex ddG approach.

Inspired by naturally occurring toxins that adopt very stable structures through multiple disulfides and/or cyclization, we utilized disulfide bridges to constrain and stabilize peptides derived from the dimerization loop as potential PPI inhibitors [31]. We truncated this loop segment (Q_120_IPQATASMKDGKWERKKFMG_140_) from the dimer interface of PHGDH (PDB code: 2G76) and subjected it to the RosettaRemodel, which offers a way of engineering disulfide bonds with minimum perturbation if a realistic disulfide bond can be made with the native backbone geometries (Figure 1a) [32]. The successes of such cyclic peptides depend on the linear precursors that conformationally pre-organize the reactive terminals in close spatial proximity before ring closure to prevent disturbance of the bioactive conformation [33,34]. This resulted in two possible site pairs, Q120-F138 and S127-E134, for inserting constraints to generate conformationally constrained peptides. We, thus, constructed a disulfide-cyclized head-to-tail peptide through positions Q120 and F138 (C_1_IPQATASMKDGKWERKKC_19_), in order to include two hot spot residues, I121 and W133, that we previously identified (Figure 1b) [30].

In addition to constraining its conformation, this cyclic peptide was also subjected to sequence optimization, as PPI interface residues with high specificity may have only modest affinity [35]. To identify affinity-enhancing mutations, we used the Rosetta flex ddG protocol, which systematically substitutes all twenty amino acid residues at each position and estimates the effects of substitutions on binding energies [36,37,38]. Before the actual redesign procedure, we replaced the hot spot I2 of the cyclic peptides (corresponding to I121 in the dimer structure) with a threonine intended to function as an N-terminal capping of the short helix, which might provide more stability than a hydrophobic residue at the beginning of a short peptide [39]. This cyclic peptide, cyc1-1 (C_1_TPQATASMKDGKWERKKC_19_), served as a starting point for the structure-based sequence optimization, which entailed calculations for each remaining position, other than the disulfide linkage, in order to get an overall view of the mutations with improved binding affinities. In the calculations, we identified Q4D, A7L/E/K, S8T/L, K10R/L/W, D11/R/N, K13S, E15Q, and R16E/Q/W as the most beneficial mutations, as compared to the native amino acid residues (Figure 1c). One or more of these putatively beneficial substitutions were introduced into the cyclic peptides, which were then synthesized for experimental evaluation.

### 2.2. The Designed Cyclic Peptides Inhibit PHGDH Activity

To evaluate the inhibitory activity of these designed cyclic peptides, we measured the enzyme activity in the presence of peptides by monitoring the NADH fluorescence (Ex 340 nM/EM 460 nM) over time using sPHGDH, which exists predominantly in a homo-dimer state with activity of about 75% of the full-length PHGDH [30]. As shown in Table 1, the peptide cyc1-1, which retained the native sequence but was constrained through head-to-tail cyclization, showed limited activity. This suggests that cyclization alone is insufficient for maintaining the peptide in its active conformation, or possibly that cyclization shifted the active conformation relative to the original segment in the homo-dimer structure. Fortunately, the peptide containing two substitutions, A7L and R16E (cyc1-2), significantly increased the activity, giving an IC_50_ of 6.4 ± 0.2 μM. Less successfully, the substitution A7K (peptide cyc1-3), or equivalently, the pair mutations of A7E and D11R (cyc1-4), which were intended to form a salt bridge interaction between positions A7 and D11 to stabilize the short helix part of the peptide, instead largely decreased or abolished the activity. In addition, another pair of mutations, K10L and R16W, combined in the peptide cyc1-5 to enhance the hydrophobic interactions with the target molecule; however, this peptide displayed only moderate activity (IC_50_ value: 13.7 ± 2.1 μM). In order to further improve the activity of peptide cyc1-2, we introduced single mutations, including Q4D, S8T, and S8L, according to our computational predictions. We found that peptides with a substitution of Q4D (cyc1-6) or S8T (cyc1-7) showed activity equivalent to peptide cyc1-2, with IC_50_ values of 5.1 ± 0.4 μM and 5.6 ± 1.2 μM, respectively, while the other variant S8L in the peptide cyc1-8 completely eliminated the activity.

Because the native sequence of the cyclic peptide consists of a large number of charged amino acid residues, we introduced polar substitutions at these positions based on our predictions, including D11N, K13S, E15Q, and R16Q, to assess the impact of the charges on the peptide activity. In particular, peptide cyc1-9 containing three such polar mutations (D11N, K13S, and E15Q) showed a promising IC_50_ value of 4.7 ± 0.7 μM, which might benefit from the balanced net charges and/or enhanced hydrogen bond interactions within the peptide. Next, we added the stabilizing substitution A7L into this peptide, which led to an inactive peptide (cyc1-10), but the activity was recovered through the combination of the point mutations A7L and S8T, as well as the retention of the native E15, as seen in peptide cyc1-11 (IC_50_ value of 5.4 ± 0.6 μM). In addition, the peptide cyc1-12 containing the substitution R16Q displayed comparable activity (IC_50_ value of 5.6 ± 1.4 μM) to peptides that have a charged amino acid at this position. These polar substitutions suggest that charges at these positions are not necessary for their activity.

To further optimize the stability of the peptide, we generated a double constrained peptide, cyc2-1 (C_1_TPQATAC_8_MKDGKWC_15_RKKC_19_), by introducing an intramolecular disulfide (C8-C15) to cyc1-1, which we hypothesized would restrict and stabilize the structural motif into its bioactive conformation [33,40]. Unlike cyc1-1, the peptide cyc2-1 with the native sequence and two constraining disulfides displayed an IC_50_ value of 12.0 ± 1.0 μM. Due to the price of ordering synthesized peptides with two constraints, we only investigated several key substitutions in peptide cyc2-1 based on our calculations of which substitutions would be most likely to improve the activity, including K10L, K10R, K10W, R16W, and R16E. We found that substitutions of K10L (peptide cyc2-2) and K10R (peptide cyc2-3) increased their activity, showing IC_50_ values of 9.4 ± 1.5 μM and 5.2 ± 1.2 μM, respectively. However, peptides with a mutation K10W (cyc2-4) or R16W (cyc2-5) significantly decreased the activities, which exhibited inhibition rates of only 44% and 27%, respectively, at the peptide concentration of 50 μM. In addition, the peptide cyc2-6 containing two mutations, including K10L and R16E, displays an IC_50_ value of 15.0 ± 1.0 μM, similar to that of peptide cyc2-1, suggesting that the effect of both substitutions might be comparable to that of the native amino acid residues. As shown in Figure S1, the inhibition dose-response curves for all the active peptides showed that the double constrained peptides require fewer substitutions to achieve a level of activity similar to the single constrained peptides.

### 2.3. The Designed Cyclic Peptides Directly Bind sPHGDH

We previously demonstrated that sPHGDH exists predominantly as a homo-dimer with a small fraction of monomers in solution [30]. In this study, we first used Microscale Thermophoresis assays (MST) to evaluate whether the active peptides found in the enzyme inhibition assay directly bind sPHGDH (Table 2).

**Figure 2:**
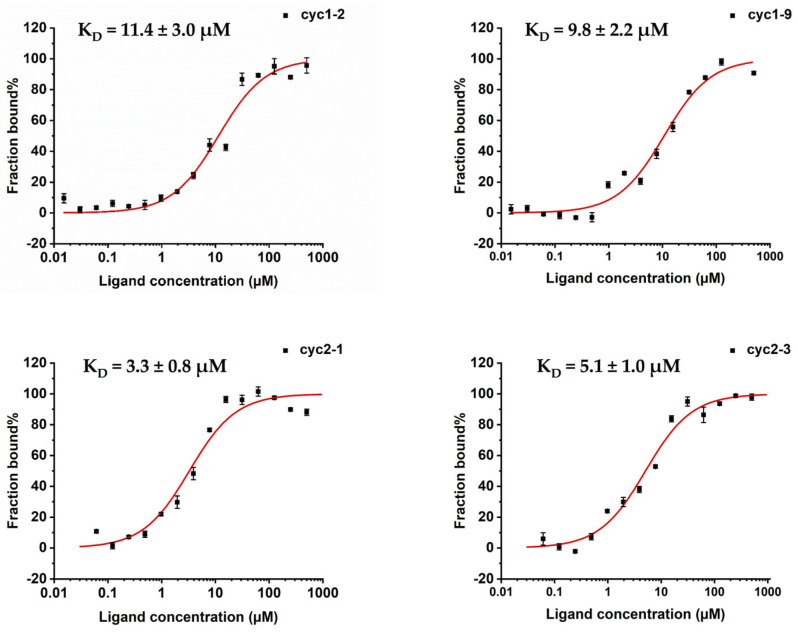
The dose-response binding curves of the representative cyclic peptides in the MST assays. Data shown represent the mean ± SD (*n* = 3).

All seven tested cyclic peptides were found to directly bind to sPHGDH in a dose-dependent manner (Table 2; the representative binding dose-response curves are shown in Figure 2 and the remaining can be found in Figure S2). Peptides constrained by two disulfides exhibited stronger sPHGDH binding potency than those peptides that are only constrained by a single disulfide bond. More specifically, head-to-tail constrained peptides in the binding assays, including cyc1-2, cyc1-6, cyc1-9, cyc1-11, and cyc1-12, showed slightly different apparent binding affinities toward the target protein, with K_D_ values of 11.4 ± 3.0 μM, 24.8 ± 7.1 µM, 9.8 ± 2.2 μM, 23.1± 4.2 µM, and 13.4 ± 3.1 μM, respectively (Table 2). Among these variant peptides, only the two non-interfacial substitutions, Q4D and S8T, in the peptides cyc1-6 and cyc1-11 showed significantly lower binding affinities (four-fold weaker than their enzymatic activities), while the others exhibited a level of binding potency comparable to their activities in the enzyme assays. The binding affinities of the double constrained peptides, including cyc2-1 and cyc2-3, are marginally more potent than their enzymatic activities, showing K_D_ values of 3.3 ± 0.8 μM and 5.1 ± 1.0 μM, respectively. According to these results, the inhibitory potencies of most measured peptides are in accordance with their binding strengths.

We further measured whether the designed cyclic peptides would affect other PPIs using three pairs of well-studied proteins, including PD1-PDL1, RXRα-PPARα, and RXRα-FXR, with SPR binding assays (Figure S5) [41,42]. As expected, these cyclic peptides did not impact the binding affinity of these three protein pairs, suggesting selectivity of the peptides.

### 2.4. The Active Cyclic Peptides Disrupt sPHGDH Dimer Formation

**Figure 3:**
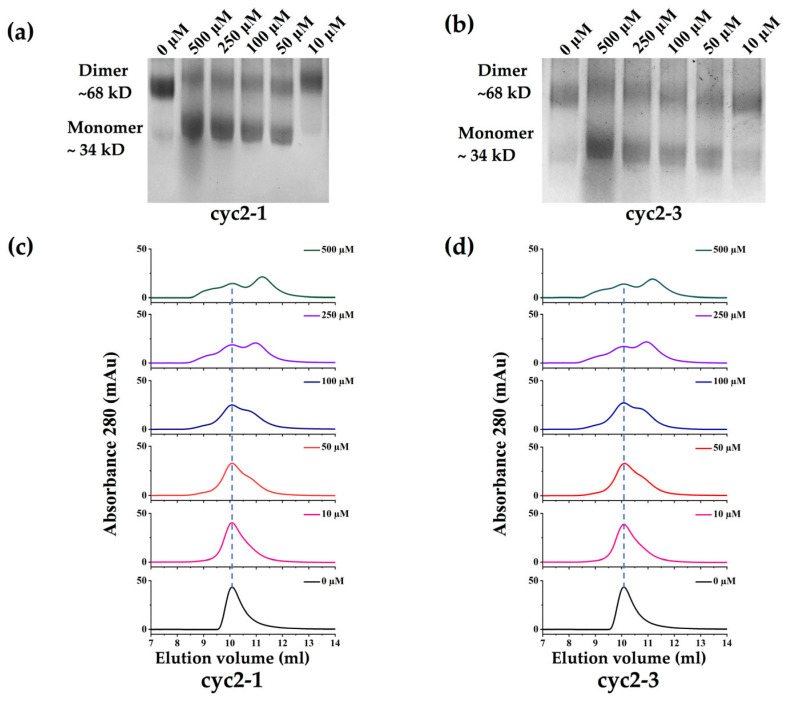
The monomer-dimer equilibrium of sPHGDH in the presence of peptides. (**a**,**b**) sPHGDH was pre-incubated with peptides (0, 10, 50, 100, 250, and 500 μM) for 30 min before chemical linking with BS3, followed by SDS/PAGE. The oligomerization state was inferred with reference to a molecular weight ladder. (**c**,**d**) Size-exclusion chromatograms (SEC) of sPHGDH in the presence of peptides (0, 10, 50, 100, 250, and 500 μM). The blue dashed line indicates the peak corresponding to the dimer form of sPHGDH.

As our peptides are designed specifically to bind to the inactive monomer and then shift the dimer-monomer equilibrium, we then measured the oligomerization state of sPHGDH in the presence of peptides using chemical cross-linking experiments. As seen in Figure 3, sPHGDH exists predominantly as a dimer with a small fraction remaining in its monomeric form when no peptide was present (0 μM). A significant dimer-disruptive effect was observed at a 10 μM concentration for the measured peptides, including cyc2-1 and cyc2-3. We also observed a dose-dependent blocking of dimer formation, with dimers and monomers existing in dynamic equilibrium. We found that decreases in the prevalence of the dimer form corresponded to proportional increases in the monomer form at increasing concentrations of peptides, ranging from 10–500 μM. At a peptide concentration of 500 μM, the oligomerization equilibrium of both cyclic peptides shifted to strongly favor the monomer state in contrast to the dimer-dominated equilibrium in the system without peptides. These results show clearly that the designed peptides bind to and stabilize a small population of the monomer, steadily increasing the prevalence of the monomeric form and eventually shifting the dimer-monomer equilibrium toward the inactive monomeric state. Together, our results suggest that cyclic-constrained peptides developed based on the loop region of the dimer interface are sufficient and effective for the inhibition of the enzyme activity of PHGDH, through directly binding to the monomeric form and disrupting the monomer-dimer equilibrium.

### 2.5. Binding Mode Analysis

**Figure 4:**
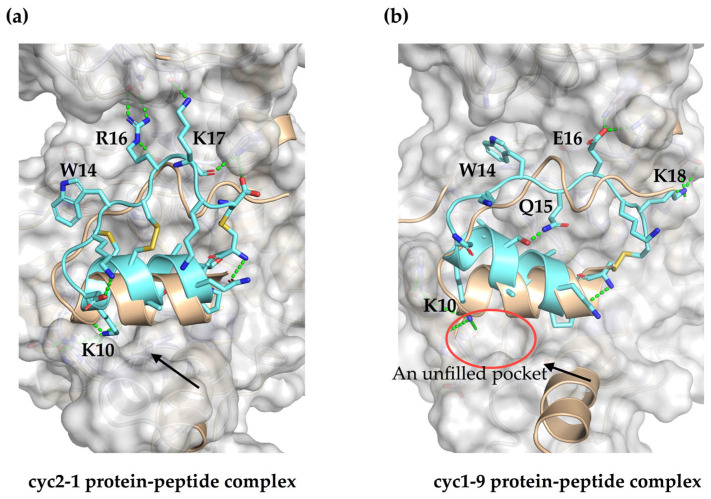
Binding mode analysis of representative single and double constrained peptides. (**a**,**b**) comparison of the binding conformation of peptide cyc2-1 (cyan) or cyc1-9 (cyan) in the protein-peptide complex extracted from MD trajectories, with the original segment (light orange) truncated from the crystal structure (PDB code: 2G76), and the binding protein surface is colored grey. Hydrogen bond interactions are shown by the green dashes, and the arrow indicates that the helix of the protein-peptide complex binds more closely than that in the homo-dimer structure. The red circle in (**b**) shows an unfilled pocket present in the protein-peptide complex.

We further investigated the binding modes of representative single and double constrained peptides with sPHGDH using molecular dynamics (MD) simulations and related analyses. As shown in Figure 4a, the intra-molecular stabilizing constraint together with cyclization ensured that peptide cyc2-1 maintained a binding conformation similar to the original segment in the homo-dimer structure, which resembles the bioactive conformation upon binding in the protein-protein complex. In contrast, cyclization alone seems to have slightly shifted the configuration of single constrained peptide cyc1-9 relative to the native segment (Figure 4b). This may explain why the binding affinities of single constrained peptides are in general weaker than those of peptides with an intramolecular disulfide. Also contributing to the decreased binding affinity, the C-terminal of the short helix part moved toward the loop region to form a more compact structure, leaving a partially unfilled pocket on the binding surface and decreased contact surfaces with the target molecule (Figure 4b). According to these results, the double constraints, rather than cyclization alone, fulfilled the goal of cyclization, which is to maintain the conformation of bioactive regions that are relevant for biological recognition and function [43,44]. Although the binding conformations of single constrained peptides are slightly different, both the single and double constrained peptides bind more closely and firmly with the target protein than the native segment, which may occur because they have less conformational flexibility [17,45,46,47]. These results suggest that constraints introduced in the flexible linear segment fix it in the bioactive (double constrained peptides) or a closely related conformation (single constrained peptides), both of which may lead to a stronger overall binding potency [40,48].

In addition, the hydrogen bond networks within the peptides or with the target are different owing to the constraining disulfides, conformational changes, or certain amino acid substitutions. For instance, positions R16 and K17 in the peptide cyc2-1 formed saltbridge or hydrogen bond interactions with D27, S28, S301, and K303 of the binding partner, while E16 and K18 in the peptide cyc1-9 interacted with K303, Q306, E304, and E122 of its counterpart instead (Figure 4a,b). According to this principle, an arginine would be a better choice of substitution for position 16 in double constrained peptides, and a corresponding glutamic acid residue would contribute equivalent interactions with the target protein for peptides that are constrained only through cyclization. Among the single constrained peptides, peptide cyc1-2 exhibited a similar but not identical binding conformation to cyc1-9, probably due to several different substitutions (A7L in cyc1-2, D11N, K13S, and E15Q in cyc1-9), which accords with their comparable levels of inhibition potency and binding affinity. These results imply that there is a large editable space for forming the same binding scaffold and binding strength using different amino acid residues and/or biomolecular interactions in protein-protein and/or protein-peptide complexes.

## 3. Discussion

Obligate homo-oligomeric proteins, such as enzymes in which the active site occurs at the subunit interface or with enzymatic activity strongly dependent on the oligomeric state, provide promising targets for designing inhibitors to bind and/or disrupt the oligomerization equilibrium [49,50]. Targeting energetically favored regions (hot spot areas) that are often associated with the α-helical or β-sheet secondary structure motifs has been a popular and valuable strategy to identify PPI modulators [51,52]. To expand the scope of druggable interfaces, we investigated the possibility of designing PPI modulators based on loop epitopes of obligate homo-oligomers, which represent one of the most challenging drug targets owing to their larger (greater than ~1500 Å^2^), more symmetric, and more hydrophobic interfaces relative to non-obligate oligomers [49,53]. Here, we demonstrated that PPI modulators can be developed by mimicking and stabilizing flexible and partially solvent-accessible loop epitopes that perturb the oligomerization equilibrium of obligate homo-oligomeric proteins. Our approach is complementary to current PPI strategies and could provide new insights into translating less druggable loop epitopes into efficient and selective oligomerization equilibrium modulators.

In this study, we designed and optimized cyclic-constrained peptides based on the loop epitope of the homo-dimer interface of PHGDH in order to harness the enhanced stability and target binding affinity of the linear peptide segment [54]. We found that these cyclic peptides, derived from loop regions, showed promising results in their inhibition potency, binding affinity, and ability to modulate the oligomerization equilibrium of PHGDH. During the optimization, we introduced interfacial substitutions to enhance side-chain interactions with the target by mimicking hot spot residues, for example, A7L, K10L, K10R, and R16E, since the side-chain atoms of these loop epitopes rarely contribute to the binding affinity [55]. In addition to interfacial mutations, we also included polar substitutions of charged amino acids to enhance hydrogen bond interactions within the peptides and/or with the target molecules, of which D11N, K13S, and E15Q, and R16Q improved the peptide activity. Our success in the current study demonstrates that cyclic peptides are a promising class of bioactive molecules potentially capable of mimicking and stabilizing loop epitopes of subunit interfaces.

Given that these cyclic peptides could effectively bind to the homo-dimer interface and disrupt the dimer formation of sPHGDH, we further measured their impacts on the oligomerization state of the full-length PHGDH, which forms a tetramer structure in solution and is expected to be more stable than the dimer form of sPHGDH [29]. To our delight, all the active peptides on sPHGDH could also inhibit the enzyme activity of PHGDH, though with slightly lowered activity (Table S1). For example, cyc1-11 and cyc1-12 are among the most active single constrained peptides on PHGDH, with IC_50_ values of 16.2 ± 1.6 μM and 20.3 ± 2.1 μM, respectively, and the corresponding IC_50_ values for double constrained peptides cyc2-1 and cyc2-3 were 37.3 ± 2.1 μM and 32.9 ± 4.0 μM, respectively (Figure S3). The remaining peptides in the PHGDH activity assay showed IC_50_ values between 30–60 μM.

These results closely accord with our previous mutagenesis study, demonstrating that interfacial mutations (for example, R119A, W133A, and I279A) have moderately or significantly weaker effects on both the enzyme activity and oligomerization equilibrium of the full-length PHGDH compared to that of sPHGDH [30]. Our studies show that the regulatory domains of PHGDH significantly contribute to the stability of the full-length structure, in addition to their key roles in oligomer formation (tetramer) and possible allosteric regulation [56,57,58]. Although non-interfacial, polar substitutions resulted in improved potency with respect to sPHGDH, we observed that only interfacial mutations directly enhancing hydrophobic interactions with the target molecule significantly increased the activities of peptides on PHGDH. In particular, peptides containing the modification A7L (including peptides cyc1-2, cyc1-6, cyc1-7, cyc1-11, and cyc1-12) showed promising inhibitory activities against PHGDH, which were probably achieved through occupying the under-utilized binding surface of the target molecule. In contrast, peptides without this mutation displayed decreased effectiveness, for example, cyc1-5, cyc1-9, as well as double constrained peptides, which are conformationally more stable but displayed lower activities compared to the single constrained peptides containing the A7L substitution. Similarly, the interfacial substitution K10L also improved the activities of peptides, such as cyc1-5 and cyc2-2, though its impact is not as strong as A7L. These results indicate that hydrophobic interactions are more important than polar interactions for disrupting tightly associated PPI interfaces, which suggests that modifications using natural and artificial amino acids to fully occupy under-utilized contact surfaces of the target molecule can serve as a potential strategy for further optimization of these constrained peptides [59,60,61].

The dimerization loop of PHGDH represents a typical example of a loop epitope located at the obligate homo-dimer interface. Its high flexibility, sequence diversity, and wide varieties of torsional angles may have a profound effect on oligomer formation and interaction specificity. Our study here, demonstrates that such loop regions provide promising templates for developing constrained peptides that inhibit PPIs through controlling the oligomerization equilibrium of oligomeric proteins. This approach can potentially be extended to other loop-mediated PPIs in oligomeric proteins, especially for those with backbone geometries that are suitable for designing macrocycles or cyclic peptides.

## 4. Materials and Methods

### 4.1. Constrained Peptides Design and Optimization

We extracted the dimerization loop (Q_120_IPQATASMKDGKWERKKFMG_140_) from the crystal structure of PHGDH (PDB code: 2G76) and scanned the backbone for suitable locations to insert constraining disulfide linkages using the RosettaRemodel [32]. Based on our results, we constructed a disulfide-cyclized head-to-tail peptide through positions Q120 and F137, and this peptide served as an initial template for sequence optimization. To identify affinity-enhancing mutations, we used the Rosetta flex ddG protocol to systematically substitute all twenty natural amino acids at each position of the peptide and estimate changes in binding free energy after mutation [36]. We followed all the recommended parameters in the flex ddG tutorial at GitHub (https://github.com/Kortemme-Lab/flex_ddG_tutorial, accessed 15 October 2019). Briefly, the protocol begins with an initial minimization for the input protein-peptide complex structure with harmonic restraints. Subsequently, the backrub method is used to generate an ensemble of models (35 models in this case) to sample local side-chain and backbone conformational changes of pivot residues. In total, 35,000 backup steps were carried out for each run as suggested. After that, the Rosetta packer is used to optimize the side-chain conformations for the wild-type and mutant sequence in parallel. Finally, another restrained minimization step is performed, before the binding free energy calculations and ΔΔG score estimations. Stabilizing substitutions are defined as those with a ΔΔG score ≤ −1.0 kcal/mol, as suggested in the tutorial. One or more of these predicted stabilizing mutations were introduced to the cyclic peptide as derivatives, which were then synthesized by a commercial company, with a purity ≥ 95%, through reverse-phase high-performance liquid chromatography (RP-HPLC) and liquid chromatography mass spectrometry.

### 4.2. Peptide Synthesis and Purification

All cyclic peptides were synthesized by GL Biochem (Shanghai, China), using a standard Fmoc-based solid-phase peptide synthesis protocol. Generally, a regioselective approach was used that involved a stepwise formation of disulfide bonds, by combining orthogonally side-chain protected cysteines (including Fmoc-Cys(trityl:Trt)-OH and Fmoc-Cys(acetamidomethyl: Acm) for the first disulfide bond between Cys(1 and 19) and the second disulfide bond between (8 and 15) respectively), and selective deprotection and oxidation of each cysteine pairs in the peptides. In brief, the first protected amino acid Fmoc-Cys(Trt)-OH was loaded to a 2-chlorotritylchloride resin and was swollen in dichloromethane (DCM) for 30 min (Step 1). After that, the resin was washed with DMF, and the Fmoc deprotection was carried out with the 20% piperidine/DMF solution for 20 min and washed again with DMF (Step 2). Next, the second Fmoc-protected amino acid, for example, Fmoc-Lys(Boc)-OH, the coupling reagent TBTU, as well as DIEA, were added to the resin and dissolved with DMF to react for about 30 min until the reaction was complete (Step 3). Steps 2 and 3 were repeated to couple other protected amino acids to the resin-peptide sequentially, according to the sequence of each peptide. Before sequential washes with DMF, DCM, and MeOH, an additional capping step of N-terminal acetylation was carried out with a solution of acetic anhydride and pyridine (1:2, *v*/*v*). Next, the protected linear peptide was cleaved from the resin and global deprotection of the side-chain protecting groups, including the Trt groups on Cys, while the Acm groups on Cys remain intact, after which it was precipitated with cold diethyl ether, washed, dried, and subjected to LC-MS analysis. The crude product was dissolved in a solution of H_2_O: MeCN (9:1, *v*/*v*), and adjusted to pH 8 with ammonium hydroxide to form the first disulfide pair (1 and 19) using air oxidation, and the Ellman test was applied to monitor the completion of the reaction. If necessary, an additional 2–3 drops of H_2_O_2_ were also added and adjusted to pH 6, then the crude peptide was subjected to LC-MS analysis and the formation of the first disulfide pair (−2 Da) was confirmed. Purification of the crude peptide using preparative HPLC created the desired cyclic peptide, as a white solid after lyophilization.

### 4.3. PHGDH Activity Assays

PHGDH activity in the presence of peptides was measured in 96-well plates (100 μL per well) at 25 °C, through monitoring the NADH fluorescence (excitation at 360 nm and emission at 460 nm) over time [62]. PAST1 and its substrate glutamate were also included in order to prevent product inhibition. In the enzyme assays, protein expression and purification, including the truncated version of PHGDH (sPHGDH, 1-307), the full-length PHGDH (1-533), and PAST1, were carried out as described in our previous papers [30,63]. To evaluate the effects of peptides on enzyme activity, the peptides were pre-incubated with enzyme samples (sPHGDH 453.3 nM or PHGDH 30.9 nM) in the assay buffer (HEPES 50 mM, pH 7.5, 10 mM MgCl_2_, and 1 mM EDTA) for 12 h. Then, 1.98 μM PAST1, 1 mM NAD^+^, and 30 mM glutamate were added and pre-incubated for 10 min before initiating the enzyme reaction via the addition of the substrate (1 mM 3-PG). Fluorescence signals were recorded for 3 min with the kinetics-reading mode of the Biotek plate reader (Synergy). IC_50_ values were obtained through fitting the data using a three-parameter Hill model of the graph of log dose against the percentage inhibition from three repeated experiments. For those peptides containing two disulfide bonds, a second step of oxidation reaction was carried out to generate the second disulfide pair. After confirming the formation of the first disulfide pair, the peptide was purified with preparative HPLC. The purified Acm-protected peptide was then treated with iodine (I_2_, 1 wt% in MeOH), dropwise under acidic conditions until the solution became brown. The crude peptide was subjected to LC-MS analysis to confirm the formation of a second disulfide pair. Purification of the crude peptide using preparative HPLC created the desired cyclic peptide, as a white solid after lyophilization.

LC-MS analysis was performed on an Agilent 6125B MC/MS system, for which 10 μL of each sample was injected on a GS-120-5-C18-BIO 4.6 × 250 mm column. Mobile phase A was 0.1% TFA in water and mobile phase B was 0.1% TFA in acetonitrile. The purity and identity of these peptides were confirmed by HPLC (>95%) and mass spectrometry (see Supplementary Materials Figure S6).

### 4.4. Microscale Thermophoresis Analysis

The binding affinity between the recombinant sPHGDH and cyclic peptides was measured with a NanoTemper Monolith NT.115 instrument (NanoTemper Technologies, Munich, Germany). sPHGDH was first adjusted to a concentration of 10 μM and then labeled with Monolith™ NT.115 Protein Labeling Kit RED-NHS (NanoTemper Technologies, Germany), according to the protocol of the manufacturer. After the protein labeling experiment was completed, sPHGDH was diluted with binding buffer (50 mM HEPES, pH 7.5, 1 mM EDTA, 10 mM MgCl2, 100 mM NaCl, and 0.005% Tween 20) to ensure that the fluorescent intensity of sPHGDH during the MST assay was about 500 RU. In this assay, the final concentration of sPHGDH after mixing with the peptide was 20 nM. Subsequently, the peptides were serially diluted in binding buffer (16 points, 1:2 dilutions, from 0.5 mM), then mixed and incubated with an equal volume of diluted sPHGDH at room temperature for 16 h. After incubation, the samples were loaded into premium treated capillaries and measured by the NanoTemper Monolith NT.115 instrument (NanoTemper Technologies, Germany). The dissociation equilibrium constant (K_D_) values were fitted by the NanoTemper Monolith affinity software MO.Affinity Analysis v2.3 (NanoTemper Technologies, Germany), using the 1:1 binding mode. 

### 4.5. Chemical Cross-Linking Experiments

sPHGDH (5 μM) was incubated with peptides (final concentrations of 0, 10, 50, 100, 250, and 500 μM) and 2.5 mM BS3 cross-linker (S5799, Sigma, Burlington, MA, USA) in the PBS buffer (40 mM, pH 7.3) at room temperature (25 °C) for 30 min. Subsequently, the reaction was quenched for 15 min through adding 500 mM Tris, with a final concentration of 45 mM. Finally, the cross-linked proteins were mixed with the loading buffer, boiled for 10 min, and then run on SDS/PAGE.

### 4.6. Size Exclusion Chromatography (SEC) Analysis

For the analysis of the sPHGDH oligomerization state by SEC, sPHGDH (20 μM) was co-incubated with peptides (final concentrations of 0, 10, 50, 100, 250, and 500 μM) in the PBS buffer (40 mM, pH 7.3) at room temperature (25 °C) for 12 h. The mixture was loaded onto a SuperdexTM 75 Increase 10/300 GL column (Cytiva, Marlborough, MA, USA) and eluted by the PBS buffer at a flow rate of 0.8 mL/min. The absorbance at 280 nm was recorded, and after each analysis the column was re-balanced by the assay buffer.

### 4.7. MD Simulations and Related Analysis

MD simulations were performed with the Gromacs 2019 package (a GPU-accelerated version), using the Amber99sb-ILDN force field to define the parameters for all the residues [64]. For each simulation, the protein-peptide complex was placed in a cubic box and intrinsic charges were neutralized using sodium or chloride ions. After 50,000 steps of the steepest descent energy minimization, each system was equilibrated using 200 ps of NPT ensemble. These equilibrated systems were simulated for 200 ns of full production MD simulations at 300 K and 1 bar barostat, with a time step of 2 fs. The atomic coordinates were recorded every 100 ps for further data analysis, and each system was repeated three times to ensure reproducibility. In addition, we extracted structures between 50–200 ns for the RMSF, clustering, molecular interaction, and binding modes analysis.

### 4.8. Surface Plasmon Resonance

The SPR binding assays were analyzed with Biacore 8K+ (Biacore (Uppsala, Sweden), Cytiva). The materials used in the SPR assays were purchased from Cytiva. PD-1 and RXRα were, respectively, immobilized on a CM5 sensor chip by using standard amine coupling at 25 °C with running buffer HBS-EP (20 mM HEPES buffer, 2.7 mM NaCl, 137 mM KCl, 1 mM EDTA, 0.05% surfactant P20, pH 7.4). The reference flow cell was activated and blocked in the absence of the protein. The immobilization level of PD-1 and RXRα were all about 1500 RU. Different concentrations of PDL1, PPARα, and FXR were serially injected into the channel to evaluate the binding affinity. Then, 5 µM cyc2-1 and cyc2-3 were, respectively, added to each concentration gradient of PDL1, PPARα, and FXR to detect the effect of the peptide on the binding affinities of PD1-PDL1, RXRα-PPARα, and RXRα-FXR.

## 5. Conclusions

Oligomerization plays an important role in protein function regulation. Modulating this process provides a promising therapeutic strategy for treating diseases with processes mediated by oligomeric proteins. Rather than targeting conserved hot spot regions, our study demonstrates that flexible loop regions can be used to design cyclic-constrained peptides for disrupting protein homo-oligomerization. The cyclic peptides discovered in this study provide a starting point for further optimization and development of PHGDH inhibitors targeting homo-oligomer interfaces.

## Figures and Tables

**Table 1:** Assessing the inhibitory activity of cyclic-constrained peptides in the PHGDH activity assays. The corresponding substitutions in each variant peptide compared to the native sequence of peptide cyc1-1 or cyc2-1 are shown in bold.

Peptide ID	Sequence	Inhibition Rate ^1^ (%)	IC_50_ Value (μM)
Peptides are only cyclization constrained			
Cyc1-1	C_1_TPQA TASMK DGKWE RKKC_19_	40%	
Cyc1-2	C_1_TPQA TLSMK DGKWE EKKC_19_		6.4 ± 0.2 µM
Cyc1-3	C_1_TPQA TKSML DGKWE EKKC_19_	19%	
Cyc1-4	C_1_TPQA TESML RGKWE EKKC_19_	None	
Cyc1-5	C_1_TPQA TASML DGKWE WKKC_19_		13.4 ± 2.0 µM
Cyc1-6	C_1_TPDA TLSMK DGKWE EKKC_19_		5.1 ± 0.4 µM
Cyc1-7	C_1_TPQA TLTMK DGKWE EKKC_19_		5.6 ± 1.2 µM
Cyc1-8	C_1_TPQA TLLMK DGKWE EKKC_19_	None	
Cyc1-9	C_1_TPQA TASMK NGSWQ EKKC_19_		4.7 ± 0.7 µM
Cyc1-10	C_1_TPQA TLSMK NGSWQ EKKC_19_	None	
Cyc1-11	C_1_TPQA TLTMK NGSWE EKKC_19_		5.4 ± 0.6 µM
Cyc1-12	C_1_TPQA TLSMK NGSWE QKKC_19_		5.6 ± 1.4 µM
Peptides with two disulfide bond linkages			
Cyc2-1	C_1_TPQA TAC_8_MK DGKWC_15_ RKKC_19_		12.0 ± 1.0 µM
Cyc2-2	C_1_TPQA TAC_8_ML DGKWC_15_ RKKC_19_		9.4 ± 1.5 µM
Cyc2-3	C_1_TPQA TAC_8_MR DGKWC_15_ RKKC_19_		5.2 ± 1.2 µM
Cyc2-4	C_1_TPQA TAC_8_MW DGKWC_15_ RKKC_19_	44%	
Cyc2-5	C_1_TPQA TAC_8_MK DGKWC_15_ WKKC_19_	27%	
Cyc2-6	C_1_TPQA TAC_8_ML DGKWC_15_ EKKC_19_		15.0 ± 1.0 µM

^1^ Inhibition rate was measured at the peptide concentration of 50 µM.

**Table 2:** Binding strength analysis of the representative peptides with sPHGDH using MST.

Peptide ID	K_D_ (μM)
Cyc1-2	11.4 ± 3.0 µM
Cyc1-6	24.8 ± 7.1 µM
Cyc1-9	9.8 ± 2.2 µM
Cyc1-11	23.1 ± 4.2 µM
Cyc1-12	13.4 ± 3.1 µM
Cyc2-1	3.3 ± 0.8 µM
Cyc2-3	5.1 ± 1.0 µM

## Data Availability

The crystal structure of PHGDH was obtained from the Protein Data Bank (RCSB PDB: https://www.rcsb.org/). We initially generated the disulfide-cyclized peptide using the RosettaRemodel (disulfide design) installed in Rosetta2018, and we identified the affinity-enhancing mutations using the Rosetta flex ddG protocol, in accordance with the flex ddG tutorial at GitHub (https://github.com/Kortemme-Lab/flex_ddG_tutorial, accessed 10 October 2018). All the MD simulations and related analyses were carried out with the GROMACS 2019 package (a GPU-accelerated version), which can be obtained at https://www.gromacs.org/. All other data generated or analyzed in this study are available from the corresponding author upon request.

## Supplementary Materials

The following supporting information can be downloaded at: https://www.mdpi.com/1420-3049/28/17/6430/s1, Figure S1: Inhibitory dose-response curves of all active peptides against sPHGDH. Figure S2: The dose-response binding curves of the additional tested cyclic peptides with sPHGDH in the MST binding assay, in addition to Figure 2. Figure S3: Backbone RMSD plots from the starting structure of peptide cyc2-1 (**a**) and peptide cyc1-9 (**b**) throughout the three independent MD simulations, respectively. Figure S4: Inhibitory dose-response curves of all active peptides in the full-length PHGDH activity assays. Figure S5: SPR sensorgrams of PD1-PDL1, RXRα-PPARα, and RXRα-FXR, with and without the presence of the cyclic peptides. Figure S6: LC-MS data on the peptides used in the PHGDH activity assays. Table S1: Inhibitory activity of the constrained peptides against the full-length PHGDH activity.

## Sample Availability

Samples of the compounds are available from the authors upon request.
