# Supplementary material for: Designing Cyclic-Constrained Peptides to Inhibit Human Phosphoglycerate Dehydrogenase
Source: Molecules. 2023 Sep 4;28(17):6430. doi: 10.3390/molecules28176430 (PMC10563079; doi:10.3390/molecules28176430)
Supplement: Supplementary file 1 [file molecules-28-06430-s001.zip › molecules-2570613-supplementary.pdf]

Supplementary Materials

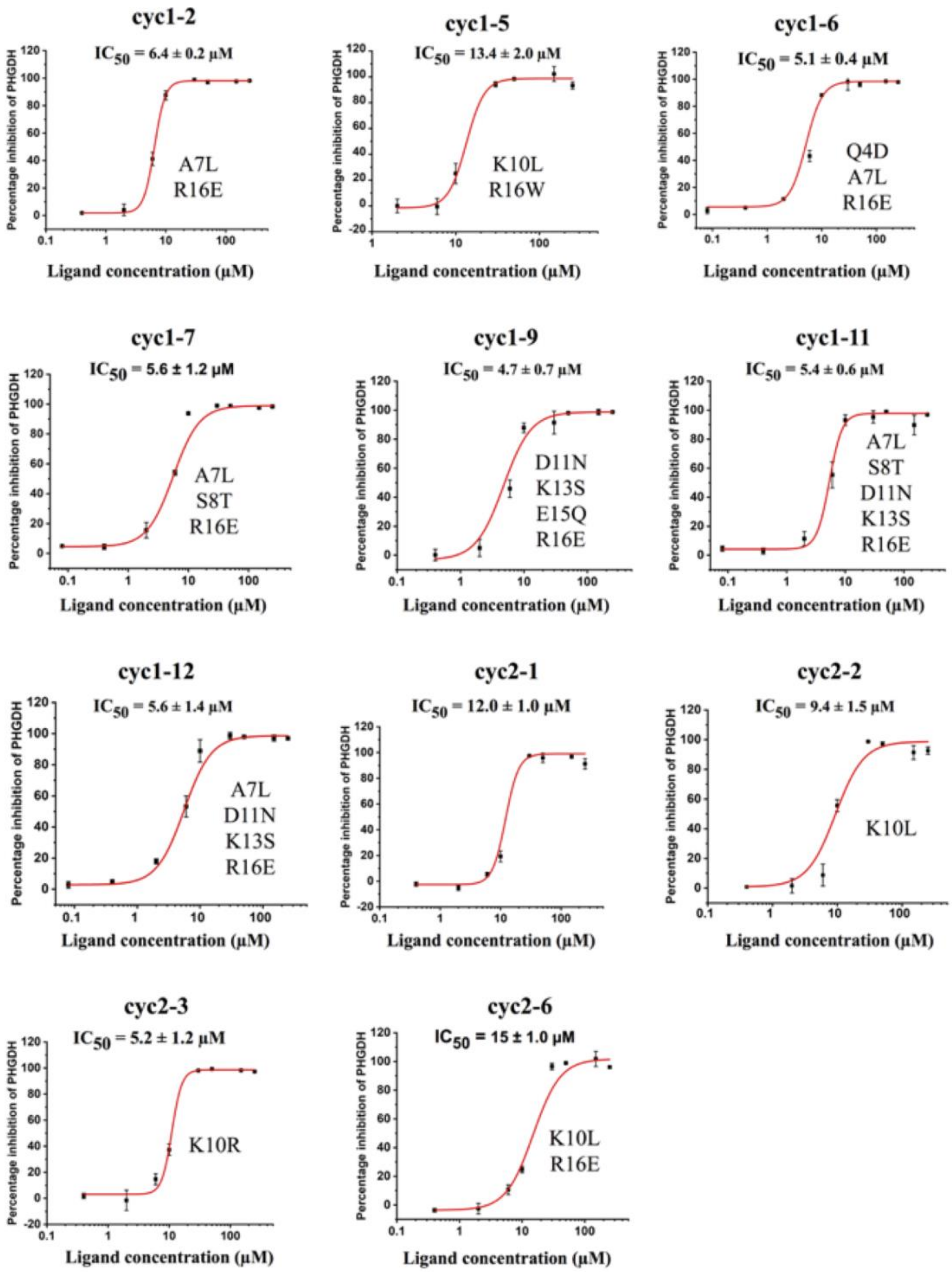

Figure S1. Inhibitory dose-response curves of all active peptides against sPHGDH. For all data, error bars represent SEM (n=3).

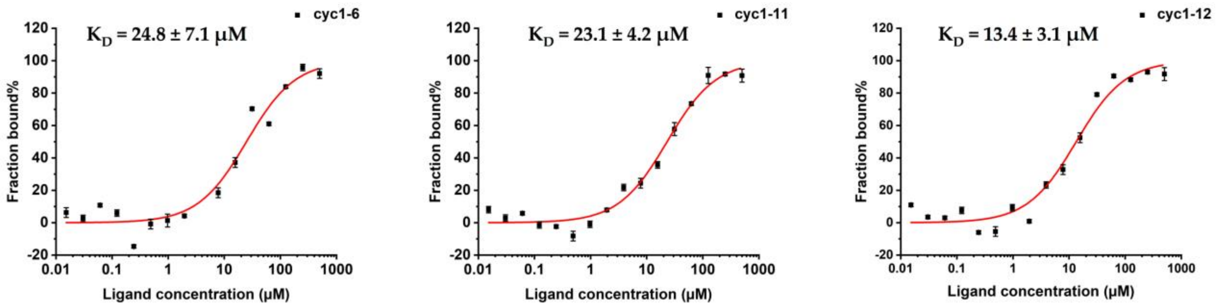

Figure S2. The affinity dose-response curves of additional tested cyclic peptides with sPHGDH in the MST binding assay in addition to Figure 2.

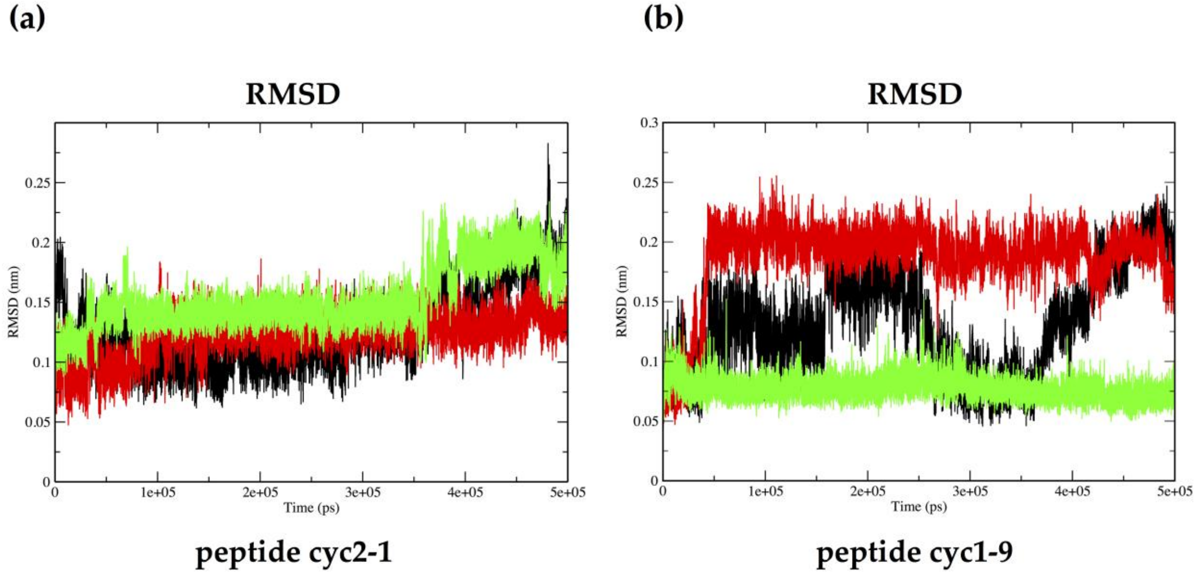

Figure S3. Backbone RMSD plots from the starting structure of peptide cyc2-1 (a) and peptide cyc1-9 (b) throughout the three independent MD simulations respectively.

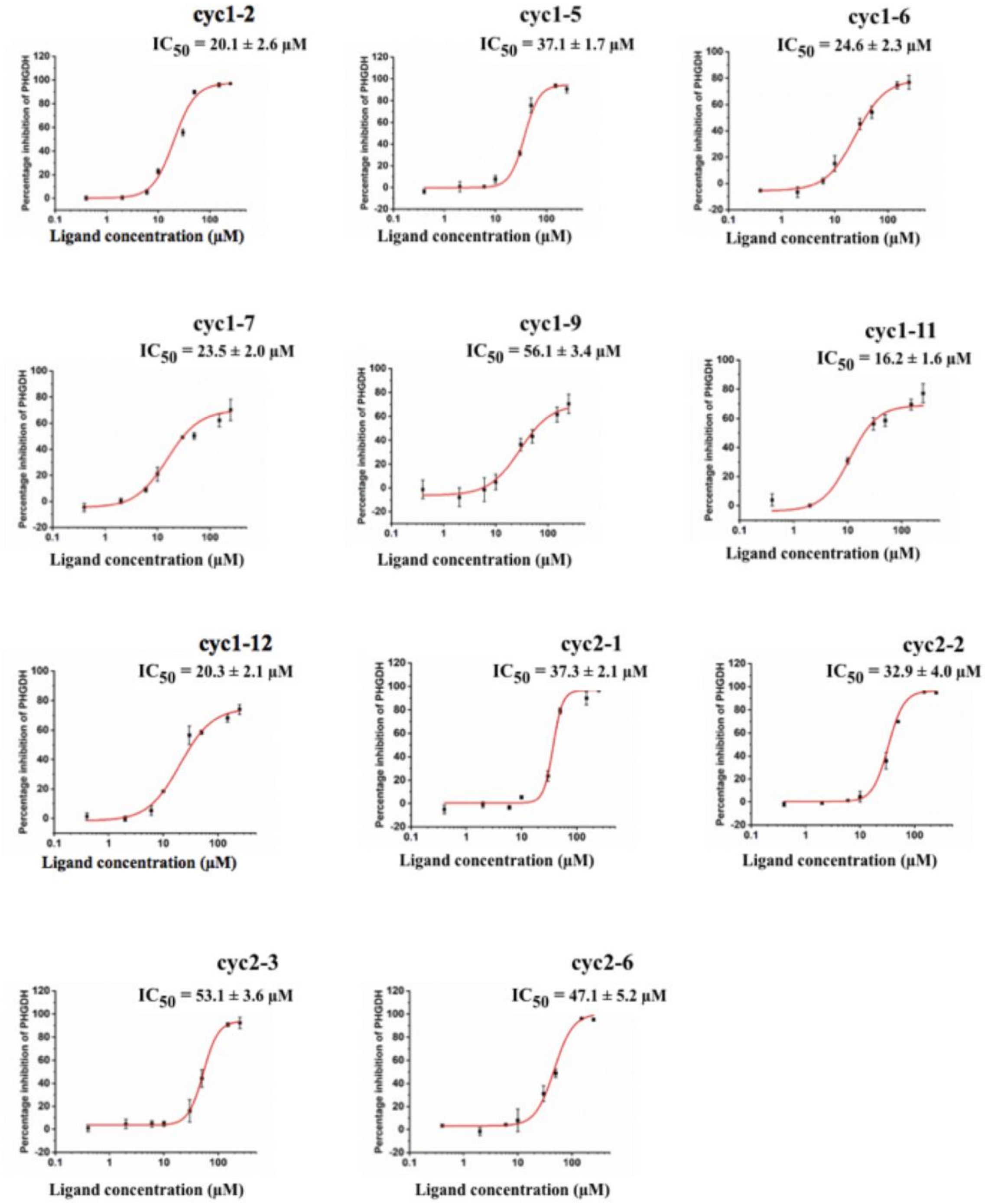

Figure S4. Inhibitory dose-response curves of all active peptides in the full-length PHGDH assays. For all data, error bars represent SEM (n=3).

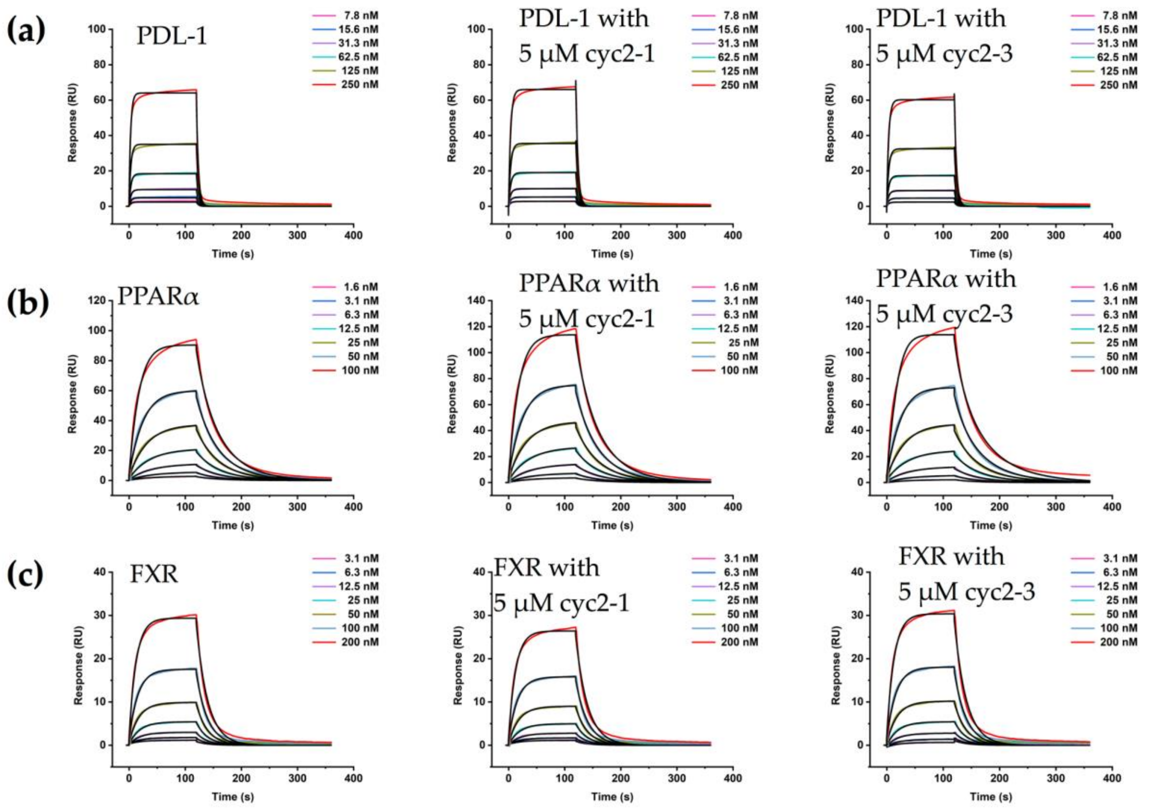

Figure S5. SPR sensorgrams of PD1-PDL1, RXRα-PPARα, and RXRα-FXR with and without the presence of the cyclic peptides. (a). SPR dose-response curves of PDL1 to PD1 in the absence or presence of 5 μM cyc2-1 or cyc2-3. (b). SPR dose-response curves of PPARα to RXRα in the absence or presence of 5 μM cyc2-1 or cyc2-3. (c). SPR dose-response curves of FXR to RXRα in the absence or presence of 5 μM cyc2-1 or cyc2-3.

Cyc1-1

Number: 0200046

Lot No:P180710-TL665489

Column: 4.6mm*250mm, Inertsil ODS-SP

Solvent A: 0.1%Trifluoroacetic in 100% Acetonirile

Solvent B: 0.1%Trifluoroacetic in 100% Water

Gradient:	A	B
0.01min	14%	86%
25.00min	39%	61%
25.01min	100%	0%
30.00min	Stop	

Flow rate:1.0ml/min

Wavelength:220nm

Volume:10 μ l

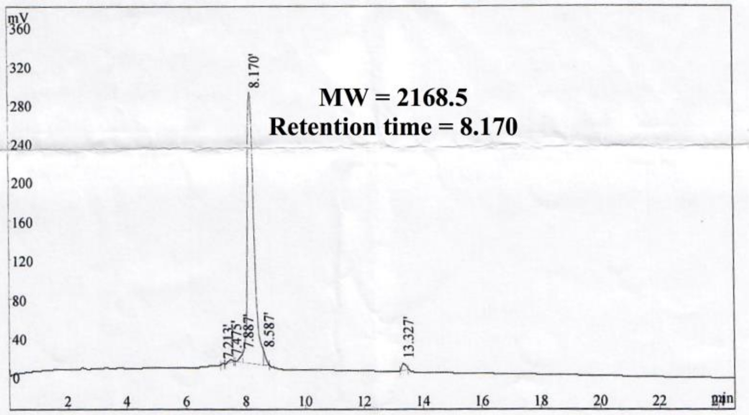

Rank	Time	Conc.	Area	Height
1	7.213	0.08154	3517	795
2	7.475	0.5144	22189	2571
3	7.887	1.26	54364	8984
4	8.170	95.11	4102068	277922
5	8.587	1.459	62944	13497
6	13.327	1.578	68067	7286
Total		100	4313149	311055

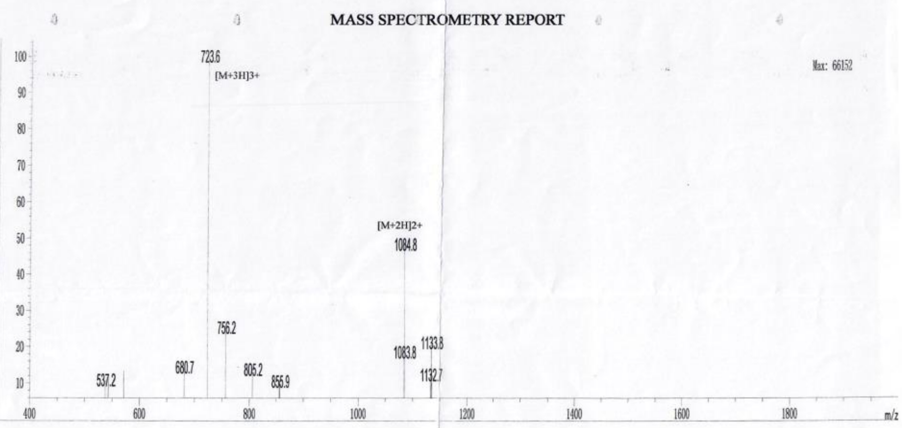

Cyc1-2

Instrument No: 0200023
Lot No :P200616-XT811448
Column :4.6*250mm, GS-120-5-C18-BIO
Solvent A :0.1%Trifluoroacetic in 100% Acetonitrile
Solvent B :0.1%Trifluoroacetic in 100% Water

Gradient :	A	B
0.00min	20%	80%
25min	45%	55%
25.1min	100%	0%
30min	Stop	

Flow rate :1.0ml/min
Wavelength :220nm
Volume :10ul

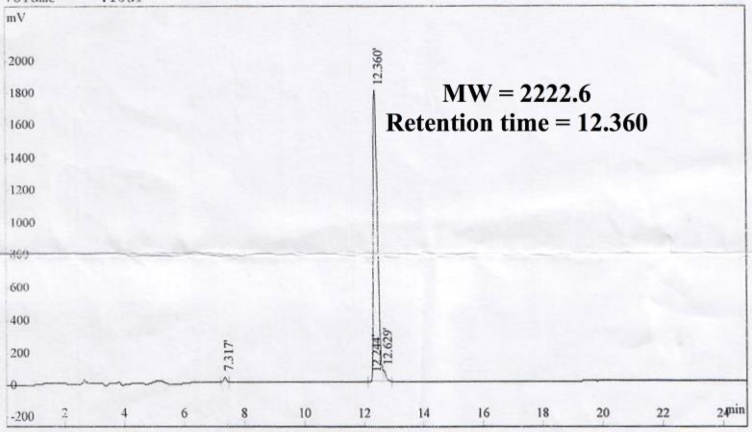

Rank	Time	Conc.	Area	Height
1	7.317	1.1576	213431	31243
2	12.244	0.1471	27113	32839
3	12.360	96.6439	17818587	1807603
4	12.629	2.0514	378228	63651
Total		100	18437359	1935336

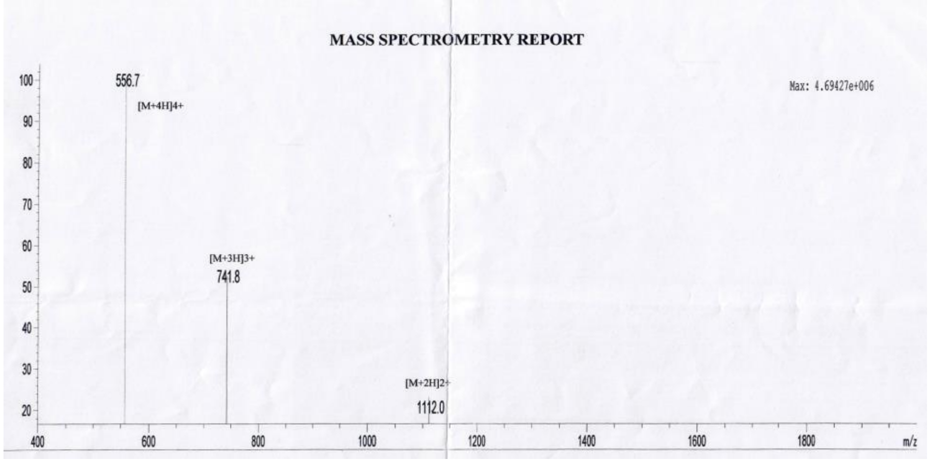

Cyc1-6

Instrument No: 0200023
Lot No :P200616-XT811451
Column :4.6*250mm, GS-120-5-C18-BIO
Solvent A :0.1%Trifluoroacetic in 100% Acetonitrile
Solvent B :0.1%Trifluoroacetic in 100% Water

Gradient :	A	B
0.00min	15%	85%
25min	40%	60%
25.1min	100%	0%
30min	Stop	

Flow rate :1.0ml/min
Wavelength :220nm
Volume :10ul

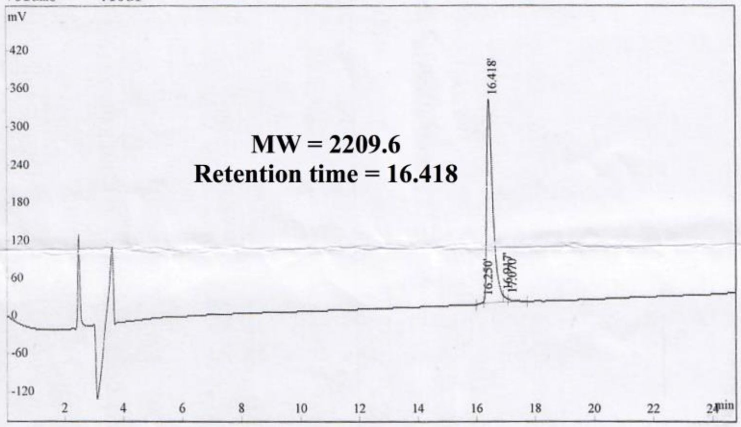

Rank	Time	Conc.	Area	Height
1	16.250	0.3860	18761	4402
2	16.418	96.6565	4697864	323378
3	16.917	1.4455	70256	10730
4	17.070	1.5119	73482	5272
Total		100	4860363	343782

MASS SPECTROMETRY REPORT

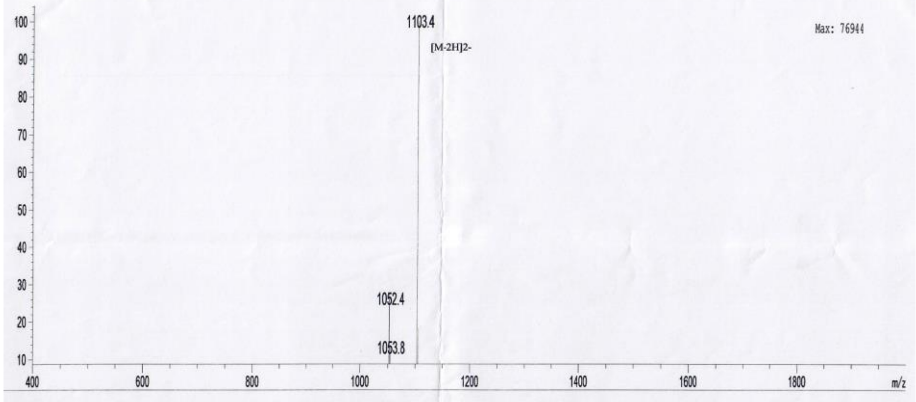

Cyc1-7

Instrument No: 0200023
Lot No :P200616-XT811455
Column :4.6*250mm, GS-120-5-C18-BIO
Solvent A :0.1%Trifluoroacetic in 100% Acetonitrile
Solvent B :0.1%Trifluoroacetic in 100% Water

Gradient :	A	B
0.00min	14%	86%
25min	39%	61%
25.1min	100%	0%
30min	Stop	

Flow rate :1.0ml/min
Wavelength :220nm
Volume :10ul

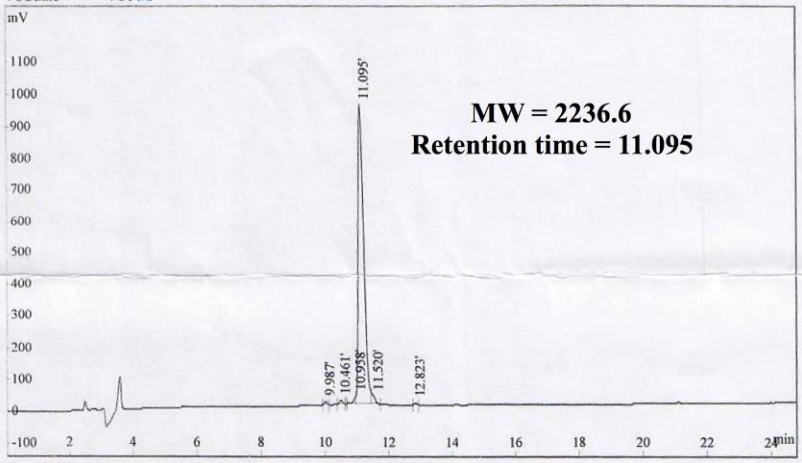

Rank	Time	Conc.	Area	Height
1	9.987	0.4944	58956	9588
2	10.461	0.6688	79741	11899
3	10.958	1.0194	121553	21756
4	11.095	95.9142	11436388	945929
5	11.520	1.7369	207098	20976
6	12.823	0.1663	19825	3698
Total		100	11923561	1013846

MASS SPECTROMETRY REPORT

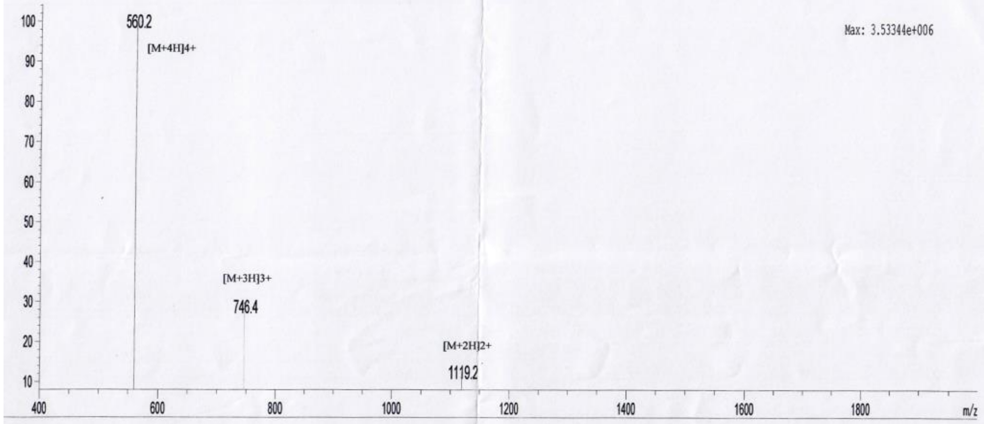

Cyc1-8

Number: 0200046

Lot No:P200616-XT811458

Column: 4.6mm*250mm, Inertsil ODS-SP

Solvent A: 0.1%Trifluoroacetic in 100% Acetonitrile

Solvent B: 0.1%Trifluoroacetic in 100% Water

Gradient:	A	B
0.01min	20%	80%
25.00min	45%	55%
25.01min	100%	0%
30.00min	Stop	

Flow rate:1.0ml/min

Wavelength:220nm

Volume:10μl

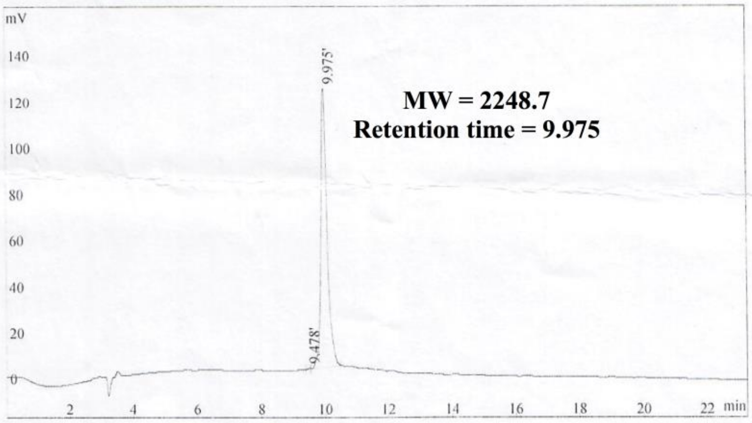

Rank	Time	Conc.	Area	Height
1	9.478	0.07107	1090	238
2	9.975	99.93	1533181	120435
Total		100	1534271	120673

MASS SPECTROMETRY REPORT

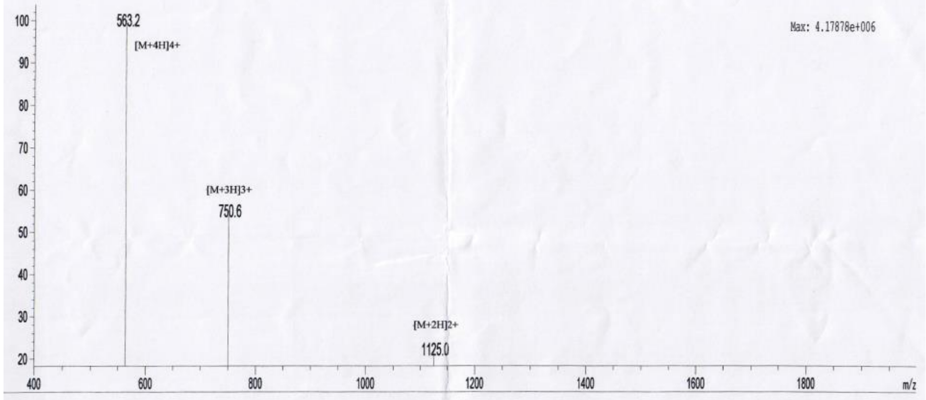

Cyc1-9

Number: 0200046

Lot No:P200616-XT811475

Column: 4.6mm*250mm, Inertsil ODS-SP

Solvent A: 0.1%Trifluoroacetic in 100% Acetonitrile

Solvent B: 0.1%Trifluoroacetic in 100% Water

Gradient:	A	B
0.01min	17%	83%
25.00min	42%	58%
25.01min	100%	0%
30.00min	Stop	

Flow rate:1.0ml/min

Wavelength:220nm

Volume:10 μ l

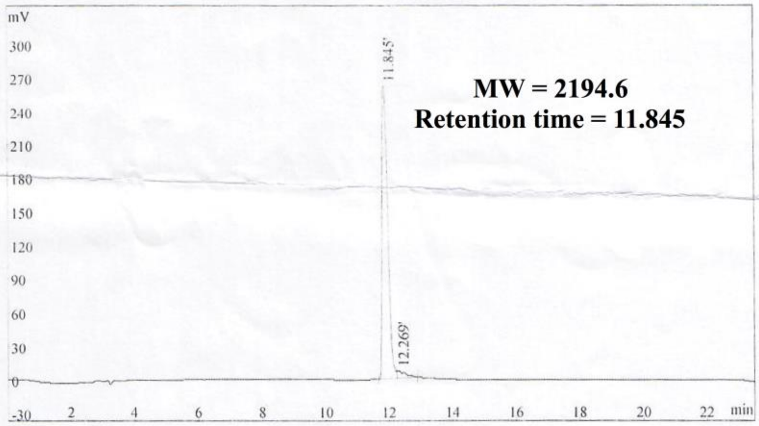

Rank	Time	Conc.	Area	Height
1	11.845	96.43	3136509	261768
2	12.269	3.57	116133	6939
Total		100	3252642	268707

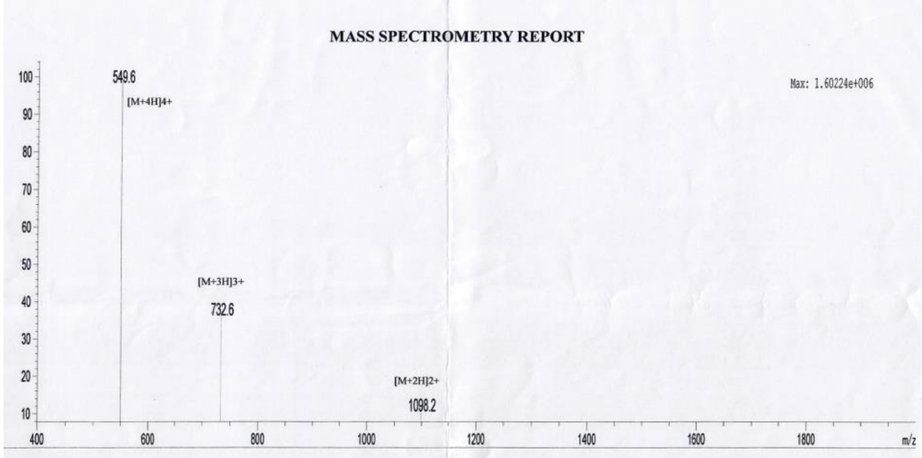

Cyc1-10

Instrument No: 0200023
Lot No :P200616-XT811429
Column :4.6*250mm, GS-120-5-C18-BIO
Solvent A :0.1%Trifluoroacetic in 100% Acetonitrile
Solvent B :0.1%Trifluoroacetic in 100% Water
Gradient :

	A	B
0.00min	12%	88%
25min	37%	63%
25.1min	100%	0%
30min	Stop	

Flow rate :1.0ml/min
Wavelength :220nm
Volume :10ul

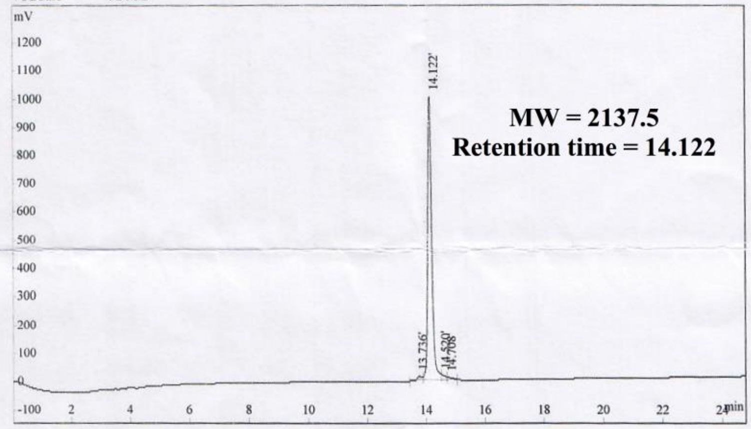

Rank	Time	Conc.	Area	Height
1	13.736	2.1458	218537	16652
2	14.122	95.1192	9687492	1020640
3	14.520	1.7088	174030	20040
4	14.708	1.0262	104517	11318
Total		100	10184576	1068650

MASS SPECTROMETRY REPORT

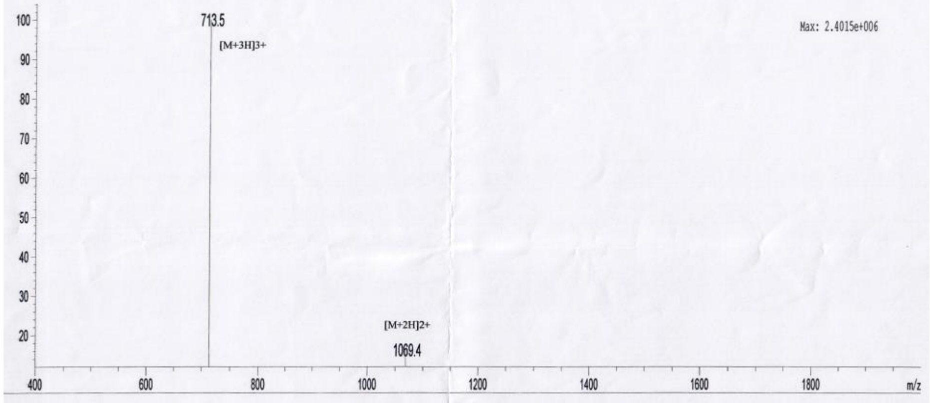

Cyc1-11

Instrument No: 0200023
Lot No :P200616-XT811433
Column :4.6*250mm, GS-120-5-C18-BIO
Solvent A :0.1%Trifluoroacetic in 100% Acetonitrile
Solvent B :0.1%Trifluoroacetic in 100% Water

Gradient :	A	B
0.00min	17%	83%
25min	42%	58%
25.1min	100%	0%
30min	Stop	

Flow rate :1.0ml/min
Wavelength :220nm
Volume :10ul

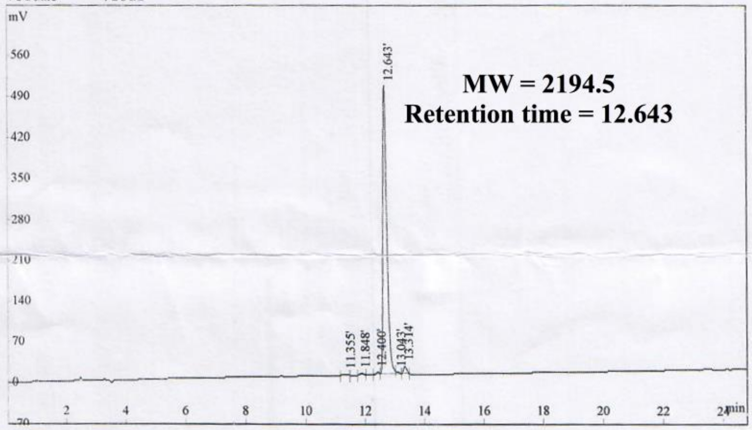

Rank	Time	Conc.	Area	Height
1	11.355	0.3569	16963	1555
2	11.848	0.4234	20122	2646
3	12.400	0.6531	31040	4434
4	12.643	95.9231	4559075	502234
5	13.043	0.6608	31408	3819
6	13.314	1.9827	94235	12431
Total		100	4752843	527119

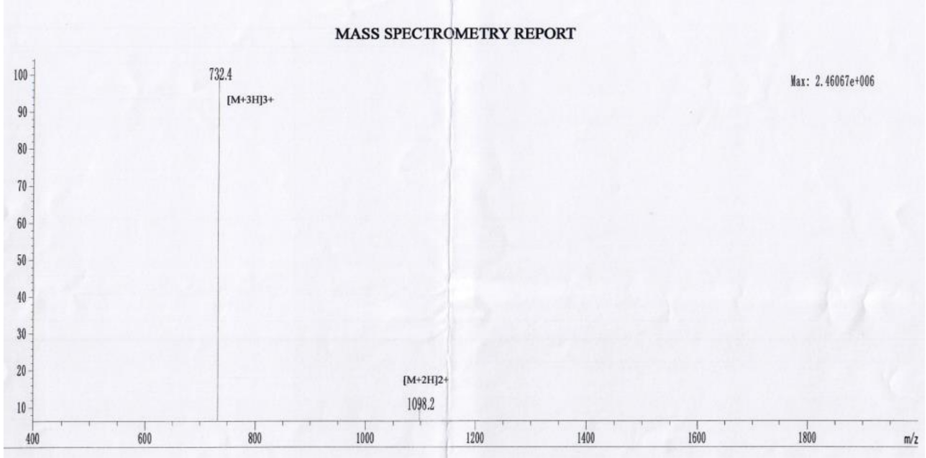

Cyc1-12

Instrument No: 0200023
Lot No :P200616-XT811444
Column :4.6*250mm, GS-120-5-C18-BIO
Solvent A :0.1%Trifluoroacetic in 100% Acetonitrile
Solvent B :0.1%Trifluoroacetic in 100% Water

Gradient :	A	B
0.00min	17%	83%
25min	42%	58%
25.1min	100%	0%
30min	Stop	

Flow rate :1.0ml/min
Wavelength :220nm
Volume :10ul

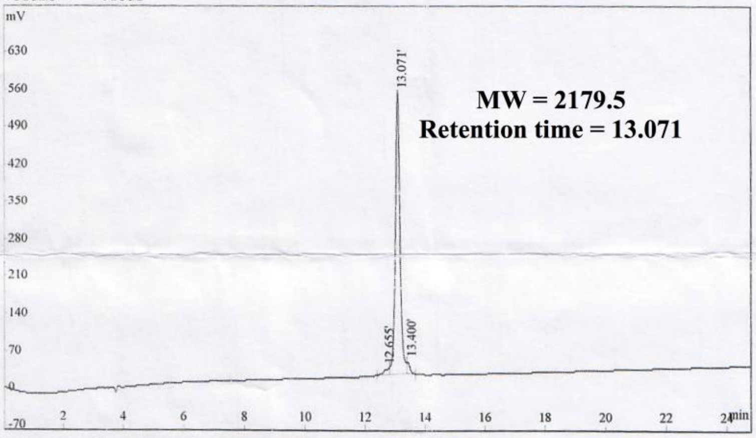

Rank	Time	Conc.	Area	Height
1	12.655	2.7565	168239	10527
2	13.071	95.2352	5812558	534763
3	13.400	2.0083	122576	21784
Total		100	6103373	567074

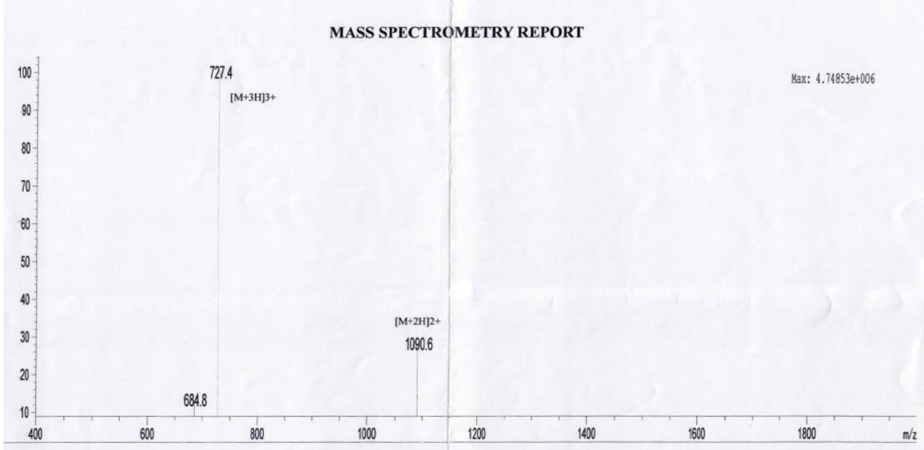

Cyc2-2

Instrument No: 0200023
Lot No :P200103-TL772662
Column :4.6*250mm, GS-120-5-C18-BIO
Solvent A :0.1%Trifluoroacetic in 100% Acetonitrile
Solvent B :0.1%Trifluoroacetic in 100% Water

Gradient :	A	B
0.00min	13%	87%
25min	38%	62%
25.1min	100%	0%
30min	Stop	

Flow rate :1.0ml/min
Wavelength :220nm
Volume :10ul

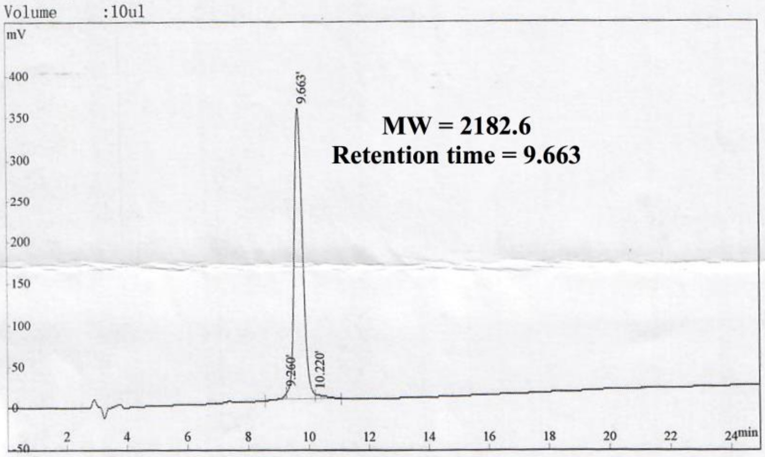

Rank	Time	Conc.	Area	Height
1	9.260	1.2379	87167	9382
2	9.663	96.9431	6826140	350510
3	10.220	1.8190	128082	5810
Total		100	7041389	365702

MASS SPECTROMETRY REPORT

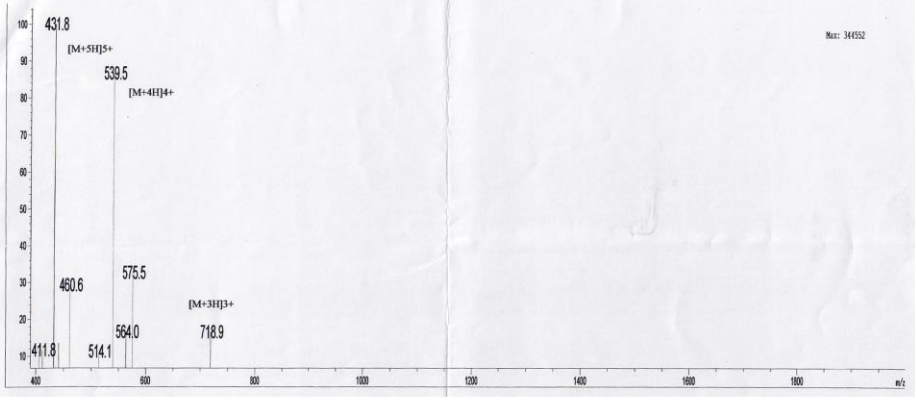

Cyc2-2

Instrument No: 0200023
Lot No :P200103-TL772662
Column :4.6*250mm, GS-120-5-C18-BIO
Solvent A :0.1%Trifluoroacetic in 100% Acetonitrile
Solvent B :0.1%Trifluoroacetic in 100% Water

Gradient	A	B
0.00min	13%	87%
25min	38%	62%
25.1min	100%	0%
30min	Stop	

Flow rate :1.0ml/min
Wavelength :220nm
Volume :10ul

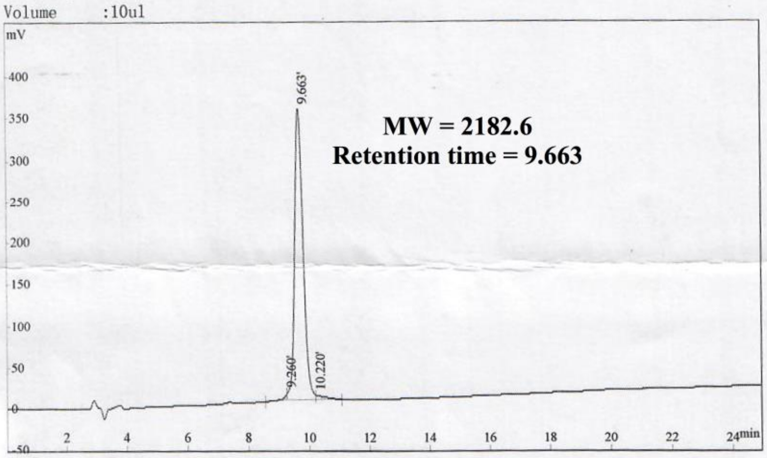

Rank	Time	Conc.	Area	Height
1	9.260	1.2379	87167	9382
2	9.663	96.9431	6826140	350510
3	10.220	1.8190	.128082	5810
Total		100	7041389	365702

MASS SPECTROMETRY REPORT

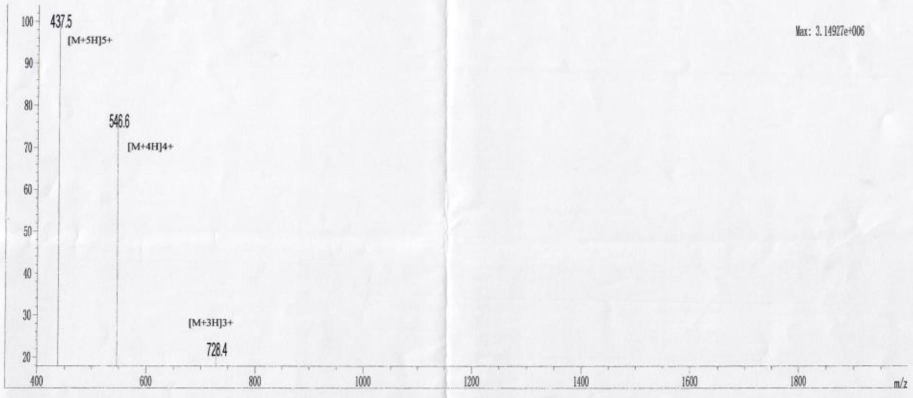

Cyc2-3

Instrument No: 0200023
Lot No :P200103-TL772663
Column :4.6*250mm, GS-120-5-C18-BIO
Solvent A :0.1%Trifluoroacetic in 100% Acetonitrile
Solvent B :0.1%Trifluoroacetic in 100% Water

Gradient :

	A	B
0.00min	20%	80%
25min	45%	55%
25.1min	100%	0%
30min	Stop	

Flow rate :1.0ml/min
Wavelength :220nm
Volume :10ul

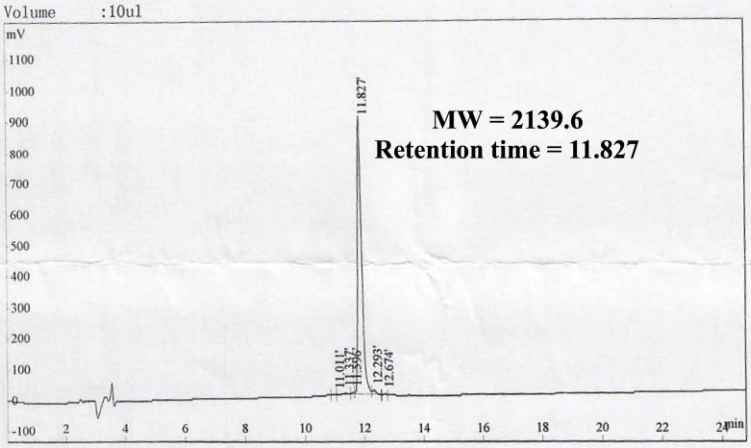

Rank	Time	Conc.	Area	Height
1	11.011	0.1886	16817	2295
2	11.337	1.1582	103251	6409
3	11.596	0.6769	60342	8157
4	11.827	96.3665	8590937	902998
5	12.293	1.4052	125274	14671
6	12.674	0.2046	18240	2798
Total		100	8914861	937328

MASS SPECTROMETRY REPORT

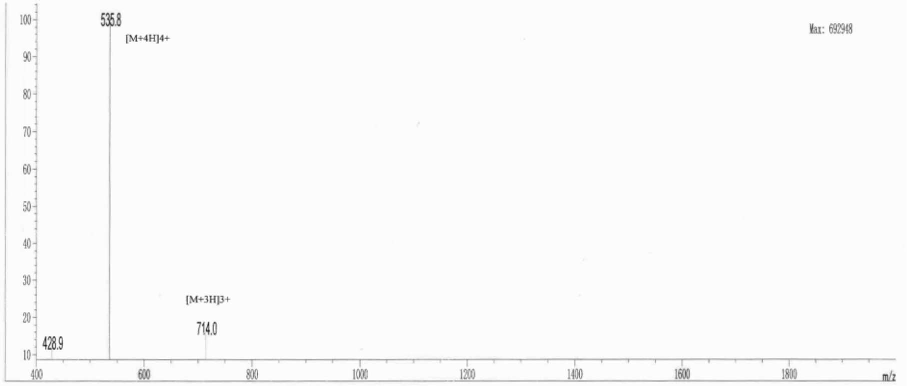

Cyc2-4

Instrument No: 0200023
Lot No :P191225-TL771175
Column :4.6*250mm, GS-120-5-C18-BIO
Solvent A :0.1%Trifluoroacetic in 100% Acetonitrile
Solvent B :0.1%Trifluoroacetic in 100% Water

Gradient :	A	B
0.00min	20%	80%
25min	45%	55%
25.1min	100%	0%
30min	Stop	

Flow rate :1.0ml/min
Wavelength :220nm
Volume :10ul

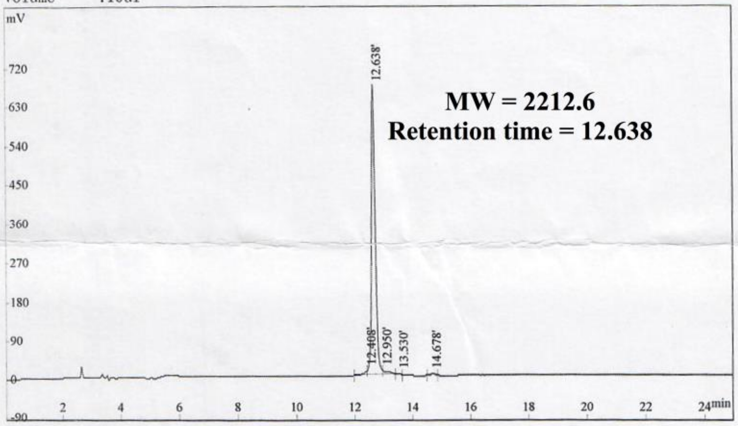

Rank	Time	Conc.	Area	Height
1	12.408	1.2668	80317	10590
2	12.638	95.9260	6081971	677156
3	12.950	1.9925	126332	9341
4	13.530	0.1759	11153	1653
5	14.678	0.6388	40499	4113
Total		100	6340272	702853

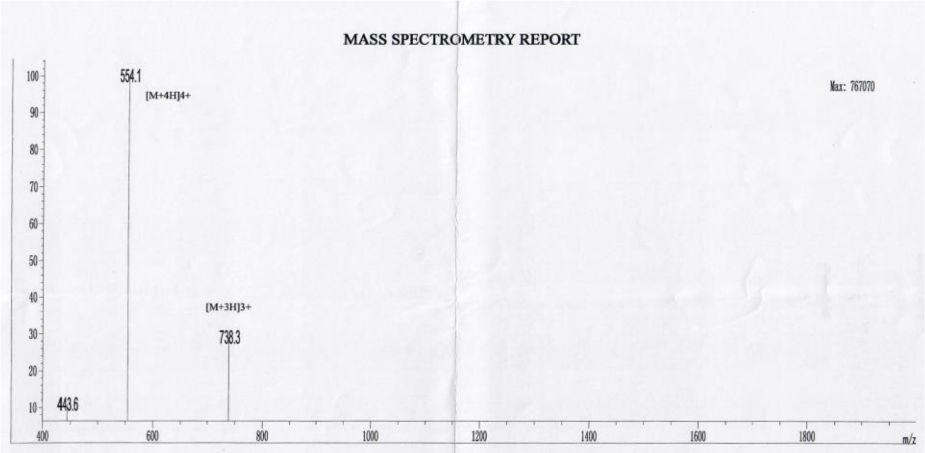

Cyc2-5

Instrument No: 0200023
Lot No :P191225-TL771176
Column :4.6*250mm, GS-120-5-C18-BIO
Solvent A :0.1%Trifluoroacetic in 100% Acetonitrile
Solvent B :0.1%Trifluoroacetic in 100% Water

Gradient :	A	B
0.00min	16%	84%
25min	41%	59%
25.1min	100%	0%
30min	Stop	

Flow rate :1.0ml/min
Wavelength :220nm
Volume :10ul

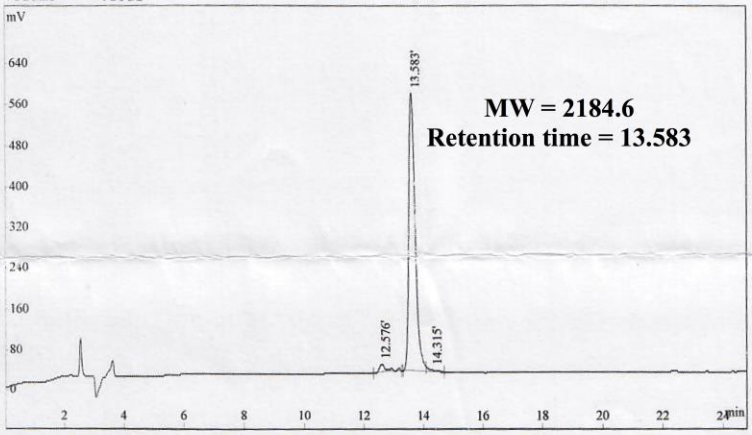

Rank	Time	Conc.	Area	Height
1	12.576	3.3809	284763	15564
2	13.583	95.5680	8049496	543936
3	14.315	1.0511	88531	2763
Total		100	8422790	562263

MASS SPECTROMETRY REPORT

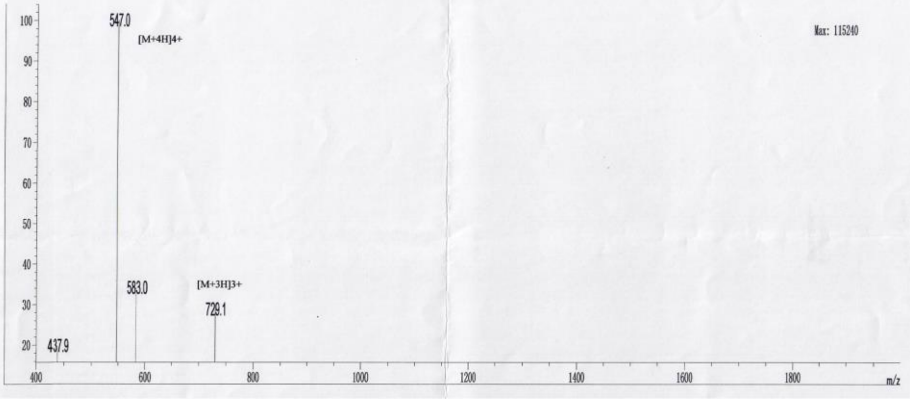

Cyc2-6

Instrument No: 0200023
Lot No :P190718-TL738839
Column :4.6*250mm, GS-120-5-C18-BIO
Solvent A :0.1%Trifluoroacetic in 100% Acetonitrile
Solvent B :0.1%Trifluoroacetic in 100% Water

Gradient :	A	B
0.00min	18%	82%
25min	43%	57%
25.1min	100%	0%
30min	Stop	

Flow rate :1.0ml/min
Wavelength :220nm
Volume :10ul

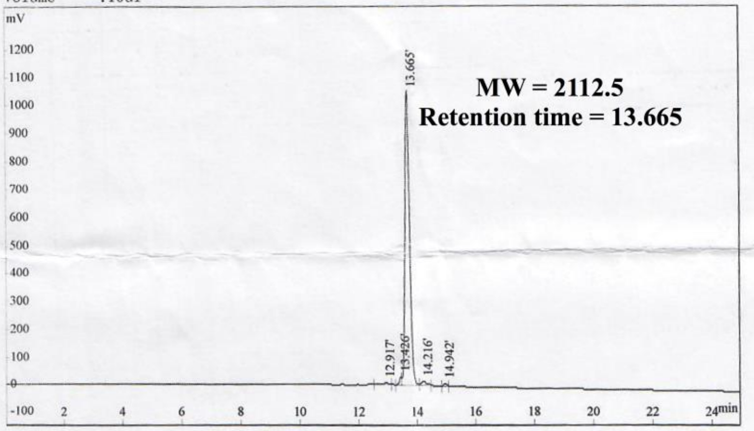

Rank	Time	Conc.	Area	Height
1	12.917	1.1397	137801	7768
2	13.426	1.5526	187731	32246
3	13.665	95.1647	11506593	1059637
4	14.216	1.6808	203227	16696
5	14.942	0.4622	55884	7734
Total		100	12091236	1124081

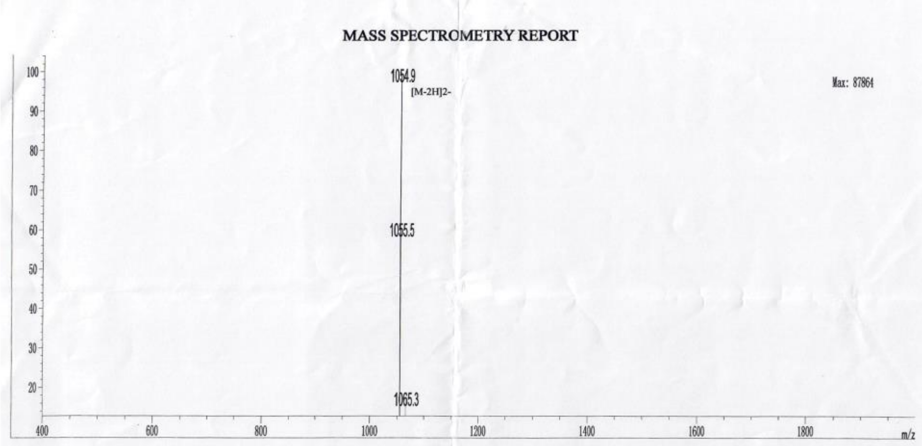

Figure S6. LC-MS data of peptides used in the PHGDH activity assays. All runs were from 5-95% at

1.0ml/min, with A = 0.1% TFA in 100% Acetonitrile, B = 0.1% TFA in 100% Water. The spectra were collected at 220 nm. Source data are provided in Source Data file.

Table S1. Inhibitory activity of the constrained peptides against the full-length PHGDH activity

Peptide ID	Sequence	IC_{50} value (μM)
Cyc1-2	C_1TPQA T**L**SMK DGKWE **E**KKC_{19}	20.1 ± 2.6 μM
Cyc1-5	C_1TPQA TASM**L** DGKWE **W**KKC_{19}	37.1 ± 1.7 μM
Cyc1-6	C_1TP**D**A T**L**SMK DGKWE **E**KKC_{19}	24.6 ± 2.3 μM
Cyc1-7	C_1TPQA T**LT**MK DGKWE **E**KKC_{19}	23.5 ± 2.0 μM
Cyc1-9	C_1TPQA TASMK **N**G**S**W**Q** **E**KKC_{19}	56.1 ± 3.4 μM
Cyc1-11	C_1TPQA T**LT**MK **N**G**S**WE **E**KKC_{19}	16.2 ± 1.6 μM
Cyc2-12	C_1TPQA T**L**SMK **N**G**S**WE **Q**KKC_{19}	20.3 ± 2.1 μM
Cyc2-1	C_1TPQA TAC_8MK DGKWC_{15} RKKC_{19}	37.3 ± 2.1 μM
Cyc2-2	C_1TPQA TAC_8M**L** DGKWC_{15} RKKC_{19}	32.9 ± 4.0 μM
Cyc2-3	C_1TPQA TAC_8M**R** DGKWC_{15} RKKC_{19}	53.1 ± 3.6 μM
Cyc2-6	C_1TPQA TAC_8M**L** DGKWC_{15} **E**KKC_{19}	47.1 ± 5.2 μM
